# Oxidized LDL-induced FOXS1 mediates cholesterol transport dysfunction and inflammasome activation to drive aortic valve calcification

**DOI:** 10.1093/cvr/cvaf159

**Published:** 2025-09-24

**Authors:** Chen Jiang, Dingyi Yao, Qiang Shen, Rui Tian, Lin Fan, Qiang Zheng, Xingyu Qian, Zongtao Liu, Yuming Huang, Nianguo Dong

**Affiliations:** Department of Cardiovascular Surgery, Union Hospital, Tongji Medical College, Huazhong University of Science and Technology, Jiefang Road, No. 1277, Wuhan, Hubei 430022, China; Department of Cardiovascular Surgery, Union Hospital, Tongji Medical College, Huazhong University of Science and Technology, Jiefang Road, No. 1277, Wuhan, Hubei 430022, China; Department of Cardiovascular Surgery, Fuwai Hospital, Chinese Academy of Medical Sciences, Fucheng Road, No. 167, Beijing 100037, China; Department of Cardiovascular Surgery, Union Hospital, Tongji Medical College, Huazhong University of Science and Technology, Jiefang Road, No. 1277, Wuhan, Hubei 430022, China; Department of Breast Surgery, Hubei Cancer Hospital, Tongji Medical College, Huazhong University of Science and Technology, Zhuodaoquan South Road, No. 116, Wuhan, Hubei 430079, China; Department of Cardiovascular Surgery, Union Hospital, Tongji Medical College, Huazhong University of Science and Technology, Jiefang Road, No. 1277, Wuhan, Hubei 430022, China; Department of Cardiovascular Surgery, The Provincial Hospital Affiliated to Shandong First Medical University, Jingwu Road, No. 324, Jinan, Shandong 250021, China; Department of Cardiovascular Surgery, Union Hospital, Tongji Medical College, Huazhong University of Science and Technology, Jiefang Road, No. 1277, Wuhan, Hubei 430022, China; Department of Cardiovascular Surgery, Union Hospital, Tongji Medical College, Huazhong University of Science and Technology, Jiefang Road, No. 1277, Wuhan, Hubei 430022, China; Department of Thoracic Surgery, The First Affiliated Hospital of Nanjing Medical University, Guangzhou Road, No. 300, Nanjing, Jiangsu 210029, China; Department of Cardiovascular Surgery, Union Hospital, Tongji Medical College, Huazhong University of Science and Technology, Jiefang Road, No. 1277, Wuhan, Hubei 430022, China

**Keywords:** Calcific aortic valve disease, Forkhead Box S1, Cholesterol transport, Inflammasome, Valvular interstitial cells

## Abstract

**Aims:**

Calcific aortic valve disease (CAVD) is becoming more prevalent with the population ageing; however, there is currently no medical therapy available. During early lipid deposition, low-density lipoprotein (LDL) mediates chronic inflammation and accelerates calcification progression. However, the mechanism still needs to be further explored.

**Methods and results:**

The study identified the transcription factor FOXS in human valvular interstitial cells (VICs) as a pivotal regulator in aortic valve calcification. Bulk RNA-seq and qRT-PCR analysis were conducted to establish that FOXS1 is induced by oxidized LDL (oxLDL) in VICs. To elucidate the role of FOXS1 in osteogenic differentiation, small interfering RNA and recombinant adenovirus were utilized to modulate FOXS1 expression in VICs. High-fat diet (HFD)-fed Apoe^−/−^Foxs1^−/−^ mice served as an *in vivo* model to investigate the role of FOXS1 in aortic valve calcification. Analysis from bulk RNA-seq, qRT-PCR, and western blot indicated significant activation of FOXS1 by oxLDL in VICs, with silencing of FOXS1 inhibiting oxLDL-induced osteogenic differentiation. Deletion of FOXS1 markedly reduced aortic valve calcification in HFD-fed Apoe^−/−^ mice, as shown by decreased calcium deposition in the aortic valve leaflets. RNA-seq and chromatin immunoprecipitation sequencing were performed to reveal the regulatory mechanisms of FOXS1, uncovering direct interactions with the promoter of BSCL2, which subsequently inhibits the expression of ABCA1 and ABCG1 via the PPARγ/LXRα axis. The study demonstrated that FOXS1 mediates VICs’ cholesterol transport dysfunction through BSCL2, ABCA1, and ABCG1 using Bodipy-cholesterol and showed that intracellular cholesterol accumulation can activate the NLRP3 inflammasome, promoting osteogenic differentiation of VICs. Additionally, it was found that IMM-H007 and recombinant BSCL2 could reduce aortic valve calcification both *in vitro* and *in vivo*.

**Conclusion:**

We identified that an oxLDL-induced transcription factor FOXS1 inhibits ABCA1 and ABCG1 expression via the BSCL2/PPARγ/LXRα axis and promotes cholesterol transport dysfunction and the activation of NLRP3 inflammasome in VICs, thereby accelerating the progression of CAVD.

## Graphical Abstract

**Graphical Abstract:**
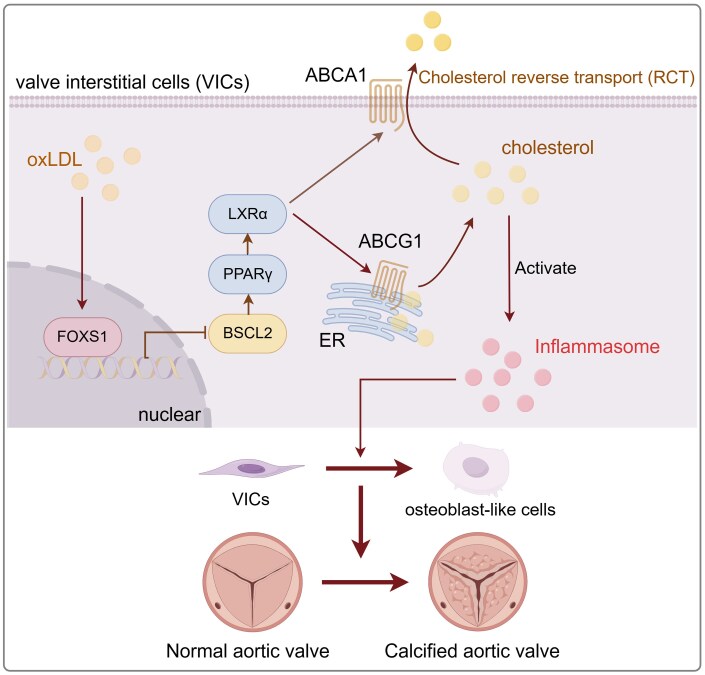
In the early stages of calcific aortic valve disease, lipid deposition occurs in the aortic valve and oxidized LDL activates FOXS1 transcription. As a transcription factor, FOXS1 inhibits BSCL2 transcription by binding to the promoter region, thereby suppressing the PPARγ–LXRα–ABC transporter axis, leading to obstructed cholesterol efflux, cholesterol accumulation in valvular interstitial cells (VICs), activation of the NLRP3 inflammasome, and promotion of osteogenic differentiation of VICs, which ultimately exacerbates valvular calcification.

**Time of primary review: 39 days**

**See the editorial comment for this article ‘oxLDL-induced aortic valve inflammation and calcification: opportunities for clinical translation ’, by Y.-J. Wang**  ***et al*****., https://doi.org/10.1093/cvr/cvaf166.**

## 1. Introduction

Calcific aortic valve disease (CAVD) is a significant cardiac valve disorder that is becoming more prevalent with the ageing population.^1^ Aortic valve replacement is currently the mainstay treatment for CAVD, yet existing prosthetic valves have inherent constraints. Consequently, there is a pressing need to investigate the underlying mechanisms of CAVD, identify potential therapeutic targets, and develop efficacious treatment approaches.

Previously, CAVD was regarded as an intricate, multifaceted, and irreversible degenerative condition. Its pathogenesis mainly includes endothelial injury, lipid accumulation, inflammatory processes, and cellular senescence.^2,3^ The subsequent pathologic progression mainly included the phenotypic transformation of valvular interstitial cells (VICs) into osteoblast-like cells. Lipid deposition has been identified as an initial indicator of calcification,^4^ with low-density lipoprotein (LDL) potentially accumulating within the aortic valve, leading to the development of chronic inflammation and facilitating osteogenic differentiation of VICs, thereby accelerating valve calcification. LDL, a key factor in cardiovascular events, has been extensively studied. Previous clinical studies have shown that statins, which lower LDL cholesterol levels, can effectively reduce the incidence of a range of cardiovascular events such as coronary heart disease, myocardial infarction, and stroke.^5,6^ However, despite substantial reductions in LDL levels, statins have limited benefits for the treatment of CAVD.^7,8^ Therefore, the association of LDL with CAVD may have its own specificity compared with other cardiovascular diseases. Elucidating the molecular mechanisms linking LDL to CAVD is crucial for clinical translation. However, natural LDL does not have a strong disease-causing capacity, and its damage is mainly due to one of its modified forms, oxidized LDL (oxLDL).^9^ Nevertheless, the specific mechanism by which oxLDL promotes VICs’ osteogenic differentiation remains unclear. Consequently, inhibiting LDL-mediated osteogenic differentiation of VICs has emerged as a crucial approach for mitigating CAVD.

The regulation of gene expression plays a crucial role in the cellular response to external signals, with transcription factors (TFs) serving as key proteins that control gene activity by binding to DNA.^10^ This regulatory mechanism is implicated in the development of various human diseases. Our hypothesis suggests that early lipid deposition may influence the activation of specific TFs, thereby modulating the processes involved in chronic inflammation and cellular osteogenic differentiation. The Forkhead box (FOX) proteins are a family of TFs, divided into 19 subfamilies from FOXA to FOXS according to their structural homology.^11^ FOXs have been reported to play an important role in the development of the heart.^12,13^ Members of the FOX protein family FOXMs,^14,15^ FOXOs,^16,17^ and FOXPs^18,19^ have been reported to play an important role in cardiovascular diseases such as cardiomyopathy, atherosclerosis, and CAVD. FOXS1, as a member of the FOX proteins family, has been reported to regulate cell proliferation, invasion, migration, and epithelial–mesenchymal transition (EMT) in a variety of cancers.^20–26^ However, the relationship between FOXS1 and cardiovascular diseases has not been reported.

Dysregulation of intracellular cholesterol is particularly important and is a hallmark of many diseases, including atherosclerotic cardiovascular disease, various cancers, and neurodegenerative diseases such as Alzheimer’s disease.^27^ ATP binding cassette (ABC) transporters are a class of ubiquitous transmembrane proteins that mediate reverse cholesterol transport (RCT) and regulate intracellular cholesterol balance. The cholesterol transporters ABC transporter A1 (ABCA1) and ABC transporter G1 (ABCG1) mediate active cholesterol efflux to apolipoprotein A1 (apoA1)^28^ and high-density lipoprotein (HDL),^29,30^ respectively. Deficiency of ABCA1 and ABCG1 in macrophages increases NLR family pyrin domain containing 3 (NLRP3) inflammasome activation and accelerates atherosclerosis in mice.^31–35^ ABCA1 is a gene mutated in Tangier disease, which is associated with low HDL levels, tissue macrophage foam cell accumulation, and possibly premature atherosclerosis.^36^ However, the relationship with CAVD and ABC transporters is unclear.

In this study, we found that in the progression of CAVD, FOXS1, as a TF activated by LDL, mediates cholesterol transport dysfunction and inflammasome activation through ABC transporters, promoting osteogenic differentiation of VICs and aortic valve calcification. This reveals previously unreported mechanisms of cholesterol accumulation and inflammatory activation in CAVD, providing new therapeutic strategies for the prevention and treatment of CAVD.

## 2. Methods

More detailed methods are described in the Supplementary material.

### 2.1 Human samples and ethics

Non-calcified aortic valves were procured from patients undergoing cardiac transplantation for dilated cardiomyopathy, while calcified aortic valves were obtained from patients undergoing aortic valve replacement for CAVD. This study adhered to the principles outlined in the Helsinki Declaration and received approval from the System Review Committee of Tongji Medical College at Huazhong University of Science and Technology. Prior to surgery, written informed consent was obtained from all participants.

### 2.2 Animal study

All animal procedures complied with the Guide for the Care and Use of Laboratory Animals published by the US National Institutes of Health (NIH publication no. 85-23, revised 1996) and were approved by the Animal Care and Use Committee of Tongji Medical College. Apoe^−/−^ and Apoe^−/−^Foxs1^−/−^ mice were purchased from GemPharmatech Co., Ltd. More information about the mice is provided in the Supplementary material.

### 2.3 Statistical analysis

Statistical analysis was performed using GraphPad Prism 8 (GraphPad Software, Inc.) and presented as mean ± standard deviation (SD). Except where otherwise noted, all quantitative studies were performed at least in six replications. The graph dots represent independent individual biological replicates. The normality of the data was confirmed using the Shapiro–Wilk test. Parametric tests, such as unpaired Student’s *t*-tests or one-way ANOVA, were employed if the data passed the normality test (alpha = 0.05). Non-parametric tests, specifically the Mann–Whitney test, were utilized if the data did not pass the normality test. The link between two variables was assessed using two-tailed Pearson correlation analysis. Statistical significance was defined as *P* < 0.05.

## 3. Results

### 3.1 Identification of oxLDL-induced FOXS1 in VICs of patients with CAVD

**Figure 1:**
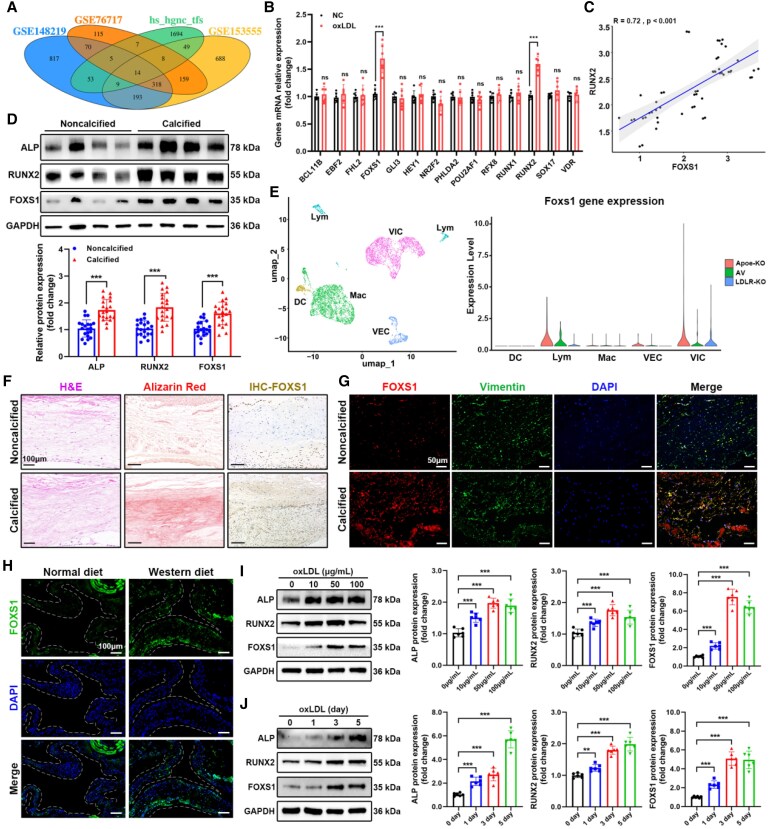
Identification of oxLDL-induced FOXS1 in VICs of patients with CAVD. (*A*) Venn diagram comparing the up-regulated DEGs in GEO datasets (GSE148219, GSE76717, and GSE153555) with human TF list. (*B*) qRT-PCR analysis of the genes in VICs treated with oxLDL. Unpaired two-tailed Student’s *t*-test (*n* = 6). (*C*) Correlation analysis between FOXS1 and RUNX2 in human aortic valves. Two-tailed Pearson correlation analysis. (*D*) Western blot of ALP, RUNX2, and FOXS1 expression in aortic valves. Unpaired two-tailed Student’s *t*-test (*n* = 20). (*E*) Uniform manifold approximation and projection (UMAP) visualization of cells in mice aortic valves and violin plot of FOXS1 expression levels in different cell types. (*F*) Alizarin Red staining and immunohistochemical staining of FOXS1 in aortic valves from CAVD patients or controls. Scale bar = 100 μm. (*G*) Immunofluorescence staining of FOXS1 and vimentin in aortic valves from CAVD patients or controls. 4′,6-diamidino-2-phenylindole (DAPI) was used for nuclear counterstaining. Scale bar = 50 μm. (*H*) Immunofluorescence staining of FOXS1 in mice aortic valves with or without HFD-fed. DAPI was used for nuclear counterstaining. Scale bar = 100 μm. (*I* and *J*) Western blot analysis of ALP, RUNX2, and FOXS1 expression in VICs treated with oxLDL at different concentrations and different time points. One-way ANOVA followed by Bonferroni multiple comparisons test (*n* = 6). Values are the mean ± SD. **P < 0.01, ***P < 0.001.

To identify the TFs activated by oxLDL in the progression of CAVD, we first compared the up-regulated differential genes from the dataset (GSE148219, GSE76717, and GSE153555) with the human TF list, resulting in 14 TFs up-regulated in the valve tissue of CAVD patients (*Figure 1A*). Subsequently, *in vitro* cell experiments were conducted in which VICs were treated with oxLDL (biological characteristics of VICs were identified in Supplementary material online, *Figures S1* and *S2*), and qRT-PCR tests revealed that, aside from RUNX family TF 2 (RUNX2), FOXS1 showed a significant activation response to oxLDL (*Figure 1B*). Interestingly, FOXS1 has a strong positive correlation with RUNX2 in three GSE datasets (*Figure 1C*). In addition, western blot analysis of collected clinical samples showed that alkaline phosphatase (ALP), RUNX2, and FOXS1 were significantly up-regulated in calcified valves (*Figure 1D*). And the up-regulation of mRNA of FOXS1 was confirmed by qRT-PCR (see Supplementary material online, *Figure S3*).

Moreover, by analysing single-cell sequencing (scRNA-seq) data of aortic valves (GSE180278), we identified five cell subtypes in the mice aortic valves like dendritic cells, lymphocytes, macrophages, valvular endothelial cells and VICs. In both models of hyperlipidaemic mice, FOXS1 expression was significantly up-regulated only in VICs (*Figure 1E*). Subsequently, histological staining of clinical samples was performed, and Alizarin Red staining confirmed the presence of significant calcium deposits in the calcified valves (*Figure 1F*), while immunohistochemical staining revealed high expression of FOXS1 in these calcified valves (*Figure 1F*). Immunofluorescence staining showed that the high expression of FOXS1 in calcified valves co-localized with the VICs’ surface marker vimentin (*Figure 1G*), further suggesting that FOXS1 plays a major role primarily in VICs. Additionally, FOXS1 was highly expressed in the aortic valves of Apoe^−/−^ mice on a high-fat diet (HFD; *Figure 1H*), which was consistent with the result of scRNA-seq.

To determine the optimal conditions for oxLDL-induced activation of FOXS1, different concentrations of oxLDL were used to treat VICs; the results showed that FOXS1 reached a high expression level when oxLDL concentration was 50 μg/mL (*Figure 1I*). Subsequent treatment of VICs with 50 μg/mL oxLDL for various durations showed that FOXS1 expression increased over time (*Figure 1J*). Interestingly, compared to other osteogenesis stimulation, FOXS1 was the most sensitive to oxLDL (see Supplementary material online, *Figure S4*). These results suggested that the activation of FOXS1 in VICs by oxLDL may be involved in the calcification process of the valves.

Given the substantial pathophysiological overlap between CAVD and vascular calcification disorders, we sought to validate the specificity of FOXS1’s association with oxLDL in VICs. Transcriptome data (GSE247718) obtained from oxLDL-treated vascular smooth muscle cells (VSMCs) were analysed, which demonstrated no significant up-regulation of FOXS1 expression following oxLDL exposure (see Supplementary material online, *Figure S5A*). To further confirm this cell type-specific response, parallel comparative experiments were performed. Consistent with the transcriptomic findings, quantitative analysis revealed that oxLDL failed to induce substantial FOXS1 activation in VSMCs (see Supplementary material online, *Figure S5B*). Notably, comparative evaluation showed that unoxidized native LDL exhibited markedly reduced capacity to activate FOXS1 compared to its oxidized counterpart (see Supplementary material online, *Figure S5B*). These collective findings suggest that LDL requires oxidative modification to acquire biological activity in this context, highlighting a critical distinction between oxidized and native LDL in FOXS1 pathway regulation.

### 3.2 FOXS1 promotes oxLDL-induced osteogenic differentiation of VICs

**Figure 2:**
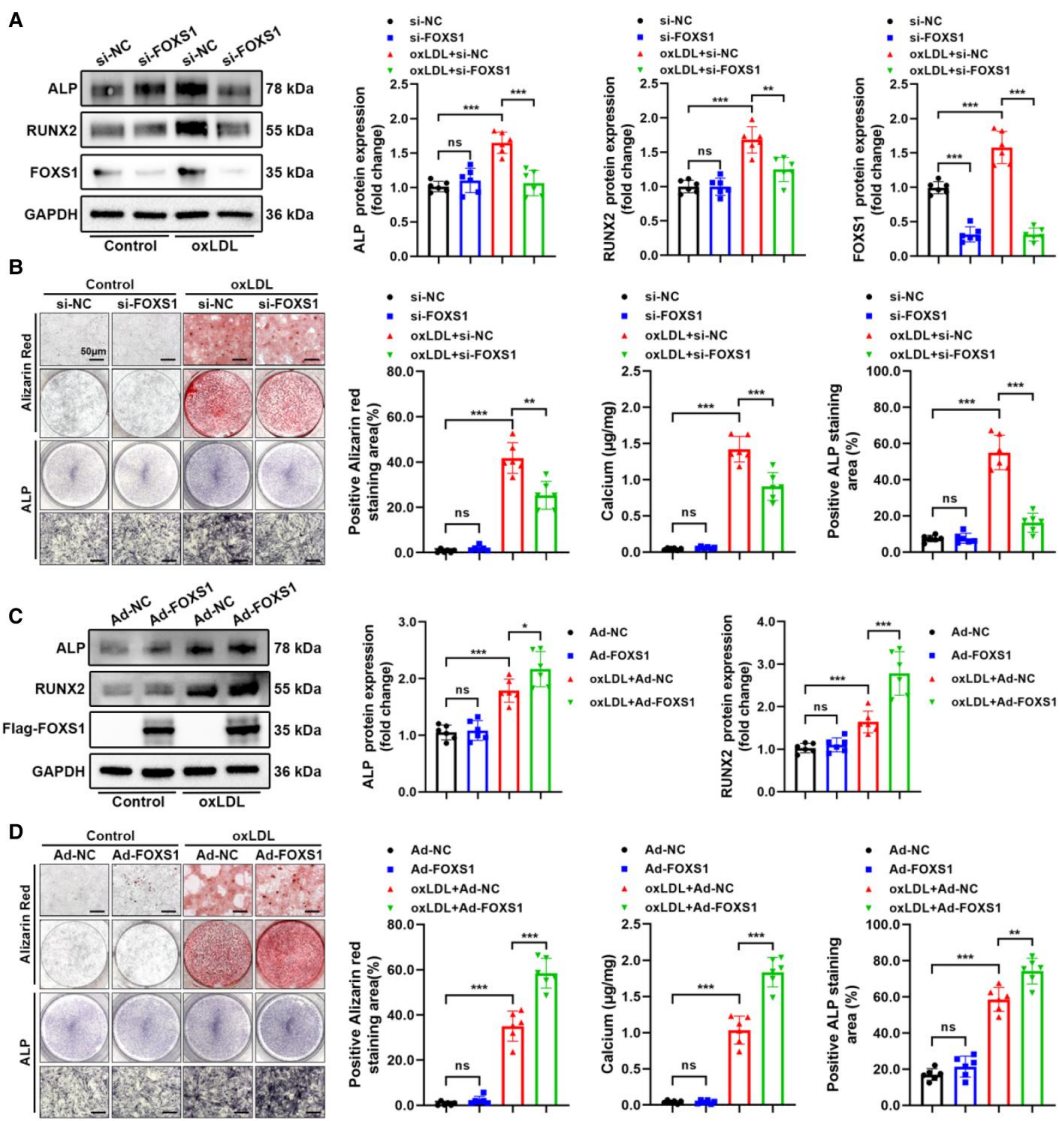
FOXS1 promotes oxLDL-induced osteogenic differentiation of VICs. (*A*) Western blot analysis of ALP, RUNX2, and FOXS1 in VICs with si-FOXS1 and oxLDL (*n* = 6). (*B*) Alizarin Red staining, calcium content, and ALP staining of VICs treated with si-FOXS1 and oxLDL (*n* = 6). Scale bar = 50 μm. (*C*) Western blot analysis of ALP, RUNX2, and Flag-FOXS1 in VICs with Ad-FOXS1 and oxLDL (*n* = 6). (*D*) Alizarin Red staining, calcium content, and ALP staining of VICs treated with Ad-FOXS1 and oxLDL (*n* = 6). Scale bar = 50 μm. Values are the mean ± SD. *P* values were calculated using one-way ANOVA followed by Bonferroni multiple comparisons test. *P < 0.05, **P < 0.01, ***P < 0.001.

To assess the impact of FOXS1 on the osteogenic differentiation of VICs, we subjected VICs to treatment with siRNA (si-FOXS1) or adenovirus (Ad-FOXS1) for a duration of 48 h. This manipulation was designed to either silence or overexpress FOXS1, respectively (see Supplementary material online, *Figure S6*, for the detection of adenovirus of FOXS1). Following this, the cells were exposed to oxLDL for varying periods: 3 days for western blot analysis, 21 days for Alizarin Red staining, and 7 days for ALP staining. The results showed that FOXS1 silencing counteracted the increased ALP and RUNX2 (*Figure 2A*), calcium deposition, and ALP level (*Figure 2B*) derived by oxLDL induction. Conversely, the overexpression of FOXS1 yielded contrasting effects (*Figure 2C* and *D*). These findings suggest that FOXS1 plays a pivotal role in enhancing the osteogenic differentiation of VICs.

### 3.3 FOXS1 deficiency attenuates aortic valve calcification *in vivo*

We successfully developed a double-knockout mouse model (Apoe^−/−^Foxs1^−/−^) and maintained them on a HFD for 24 weeks to induce aortic valve calcification *in vivo*. The successful knockout of FOXS1 was validated through immunofluorescence staining, providing a clear indication of the absence of FOXS1 expression in these mice (see Supplementary material online, *Figure S7*).

**Figure 3:**
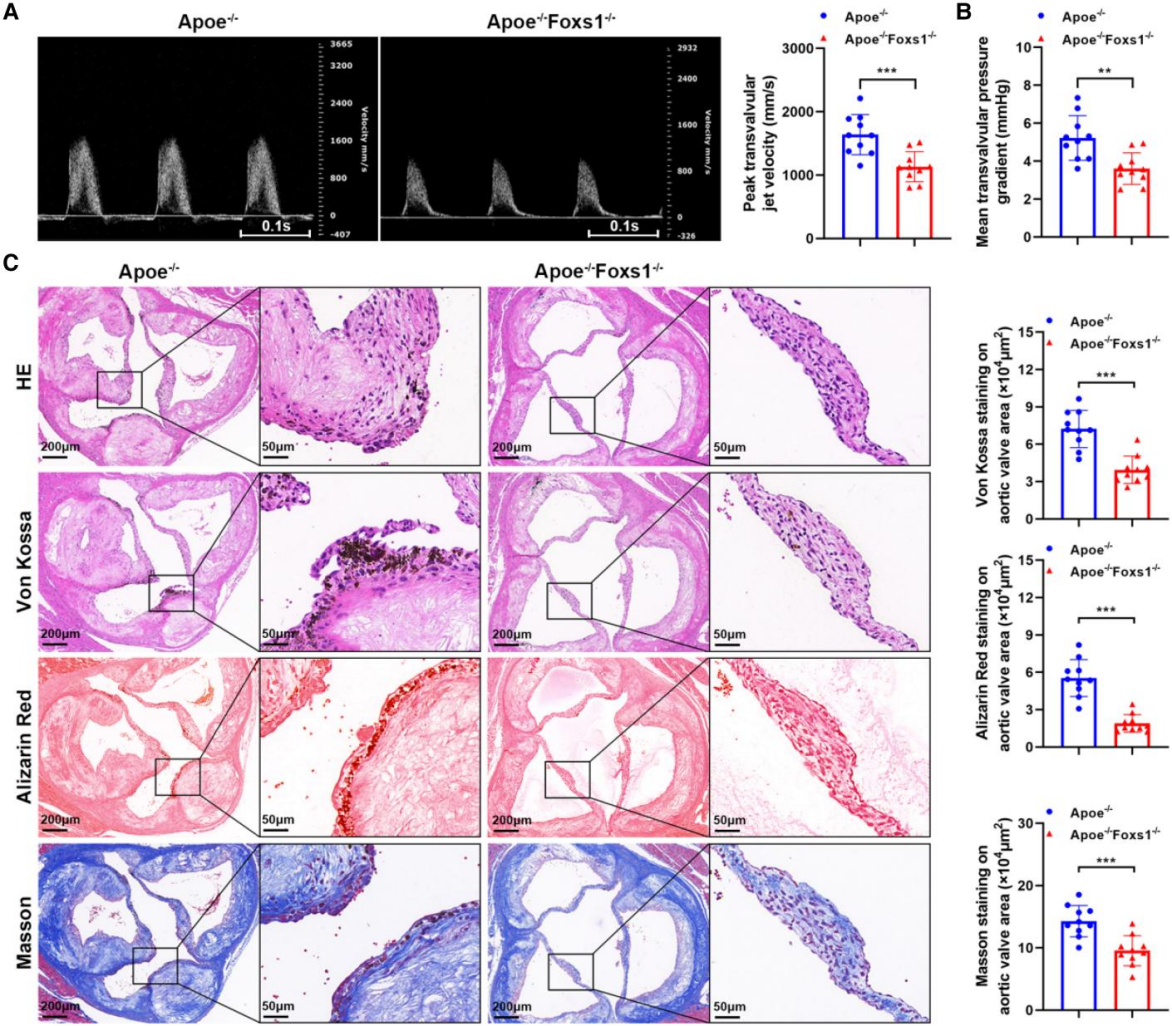
FOXS1 deficiency attenuates aortic valve calcification *in vivo*. (*A* and *B*) The severity of aortic valve stenosis in Apoe^−/−^ and Apoe^−/−^Foxs1^−/−^ mice fed with HFD was evaluated using echocardiography. Pulsed-wave Doppler examination was conducted across the aortic valve to measure peak transvalvular jet velocity (*A*) and the mean transvalvular pressure gradient (*B*). (*C*) HE, Von Kossa, Alizarin Red, and Masson’s staining of aortic valves from Apoe^−/−^ and Apoe^−/−^Foxs1^−/−^ mice fed with HFD. Values are the mean ± SD. Data were analysed by unpaired two-tailed Student’s *t*-test (*n* = 10). **P < 0.01, ***P < 0.001.

After 24 weeks of feeding on a HFD, there were no significant differences in the basic conditions of the mice (see Supplementary material online, *Table S3*). To determine whether the deficiency of FOXS1 affects the calcification of the aortic valve in mice, ultrasonic measurement of the aortic valve flow velocity was used. The results showed that mice with FOXS1 deficiency had significantly lower aortic valve flow velocity and mean aortic valve pressure gradient (*Figure 3A* and *B*), but there was no significant difference in cardiac function (see Supplementary material online, *Table S3*). Furthermore, Apoe^−/−^Foxs1^−/−^ mice had reduced aortic valve calcification compared with Apoe^−/−^ mice, as evidenced by Von Kossa, Alizarin Red, and Masson’s staining (*Figure 3C*). These results indicated that FOXS1 deficiency attenuated aortic valve calcification *in vivo*.

### 3.4 FOXS1 mediates osteogenic differentiation of VICs by inhibiting ABCA1 and ABCG1

**Figure 4:**
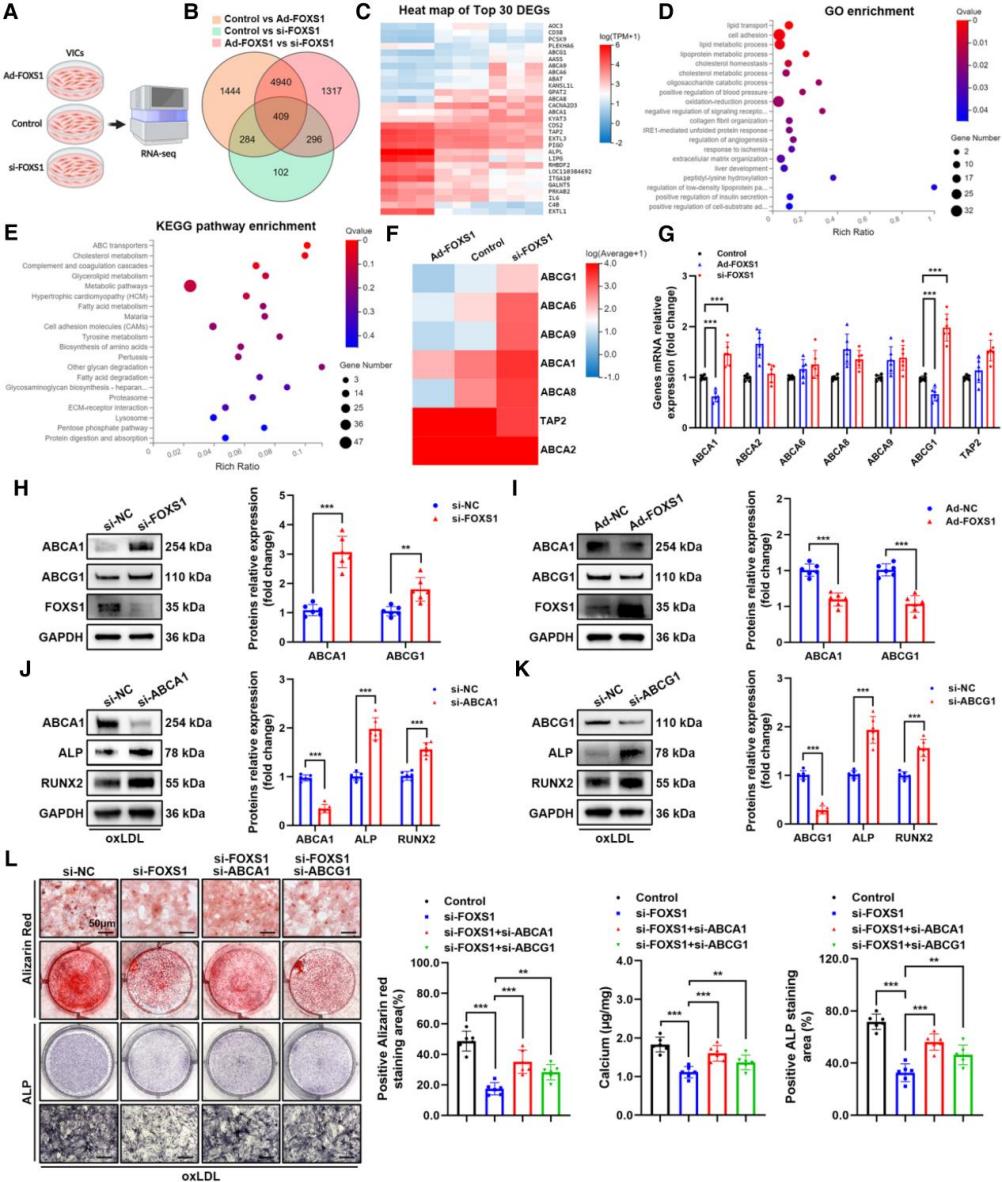
FOXS1 mediates osteogenic differentiation of VICs by inhibiting ABCA1 and ABCG1. (*A*) Schematic of RNA-seq for VICs treated with Ad-FOXS1, si-FOXS1, and negative control. (*B*) Venn diagram interaction of DEGs of the three groups compared with each other and 409 common DEGs were found. (*C*) Heatmap showing the top 30 DEGs in the 409 common DEGs. (*D* and *E*) GO enrichment (*D*) and KEGG enrichment (*E*) of common DEGs. The size of the bubbles corresponds to the number of genes that align with the enrichment, and the rich ratio reflects the count of genes that match within the pool of integrated background genes. (*F*) Heatmap showing the DEGs of ABC transport in KEGG pathway enrichment. (*G*) qRT-PCR analysis of the genes in VICs treated with Ad-FOXS1, si-FOXS1, and negative control (*n* = 6). (*H–K*) Western blot analysis of protein levels in VICs (*n* = 6) treated with si-FOXS1 (*H*), Ad-FOXS1 (*I*), si-ABCA1 (*J*), and si-ABCG1 (*K*). (*L*) Alizarin Red staining, calcium content, and ALP staining of VICs treated with si-FOXS1 and si-ABCA1 or si-ABCG1 (*n* = 6). Scale bar = 50 μm. Values are the mean ± SD. *P* values were calculated using unpaired two-tailed Student’s *t*-test (*G–K*) or one-way ANOVA followed by Bonferroni multiple comparisons test (*L*). **P < 0.01, ***P < 0.001.

To explore the underlying mechanisms through which FOXS1 influences the osteogenic differentiation of VICs, we conducted RNA sequencing on VICs, which were divided into three groups: (i) Ad-FOXS1 (si-NC +Ad-FOXS1), (ii) si-FOXS1 (si-FOXS1 + Ad-NC), and (iii) control (si-NC+ Ad-NC). The diagram and data of RNA-seq were shown in *Figure 4A* and Supplementary material online, *Excel File S1*. We compared the differential genes between each pair of conditions and identified 409 common differential genes (*Figure 4B*). The top 30 significant differentially expressed genes (DEGs) were displayed in a heatmap (*Figure 4C*). Gene ontology (GO) enrichment analysis of these 409 DEGs revealed that FOXS1 significantly affects lipid transport, lipid metabolism, and cholesterol metabolic processes in VICs (*Figure 4D*). Kyoto encyclopedia of genes and genomes (KEGG) pathway enrichment demonstrated significant impacts on ABC transporters and cholesterol metabolism (*Figure 4E*). ABC transporters, a class of transmembrane transport proteins, have been reported to mediate RCT in macrophages, reduce foam cell formation, and alleviate atherosclerosis, but not yet mentioned in CAVD and VICs. We hypothesize that ABC transporters may be key to FOXS1-regulated cholesterol transport and lipid metabolism in VICs. The KEGG enrichment analysis included seven genes related to ABC transporters: ABCG1, ABCA6, ABCA9, ABCA1, ABCA8, TAP2, and ABCA2 (*Figure 4F*). qRT-PCR analysis revealed a stable negative regulatory relationship between ABCA1, ABCG1, and FOXS1 in VICs (*Figure 4G*). This regulatory relationship was further confirmed in a western blot analysis (*Figure 4H* and *I*). Besides, the immunofluorescent staining showed increased expression of Abca1 and Abcg1 in the aortic valve leaflets of Foxs1^−/−^ mice (see Supplementary material online, *Figure S8*). These results all indicate that FOXS1 can suppress the expression of ABCA1 and ABCG1.

To investigate whether ABCA1 and ABCG1 affect the osteogenic differentiation of VICs, we silenced these two genes using siRNA and then treated VICs with oxLDL. Western blot analysis showed that silencing either ABCA1 or ABCG1 up-regulated ALP and RUNX2 expression (*Figure 4J* and *K*), reversing the inhibitory effect of FOXS1 silencing on osteogenic differentiation of VICs (*Figure 4L*). These findings suggest that FOXS1 influences oxLDL-induced osteogenic differentiation of VICs through ABCA1 and ABCG1. Interestingly, ABCA1 seems to play a more crucial role than ABCG1, possibly related to their cellular localization. ABCG1 is mainly localized in the endoplasmic reticulum and Golgi apparatus membranes, whereas ABCA1 is primarily located at the plasma membrane, thus directly affecting intracellular cholesterol levels.^37^

### 3.5 Cholesterol transport dysfunction drives NLRP3 inflammasome activation and osteogenic differentiation in VICs

The absence of ABC transporters can lead to cholesterol accumulation within macrophages, thereby activating NLRP3 inflammasome and exacerbating the formation of atherosclerotic plaques.^35^ The NLRP3 inflammasome has shown potential as a therapeutic target for CAVD.^38–40^ The above results have proven that FOXS1 can mediate osteogenic differentiation of VICs induced by oxLDL through ABC transport proteins. However, the role of ABC transporters or cholesterol in CAVD disease has not yet been reported. We hypothesize that a decrease in ABC transporter expression mediated by FOXS1 in VICs leads to impaired cholesterol transport and intracellular cholesterol accumulation and promotes NLRP3 inflammasome activation and osteogenic differentiation in VICs.

**Figure 5:**
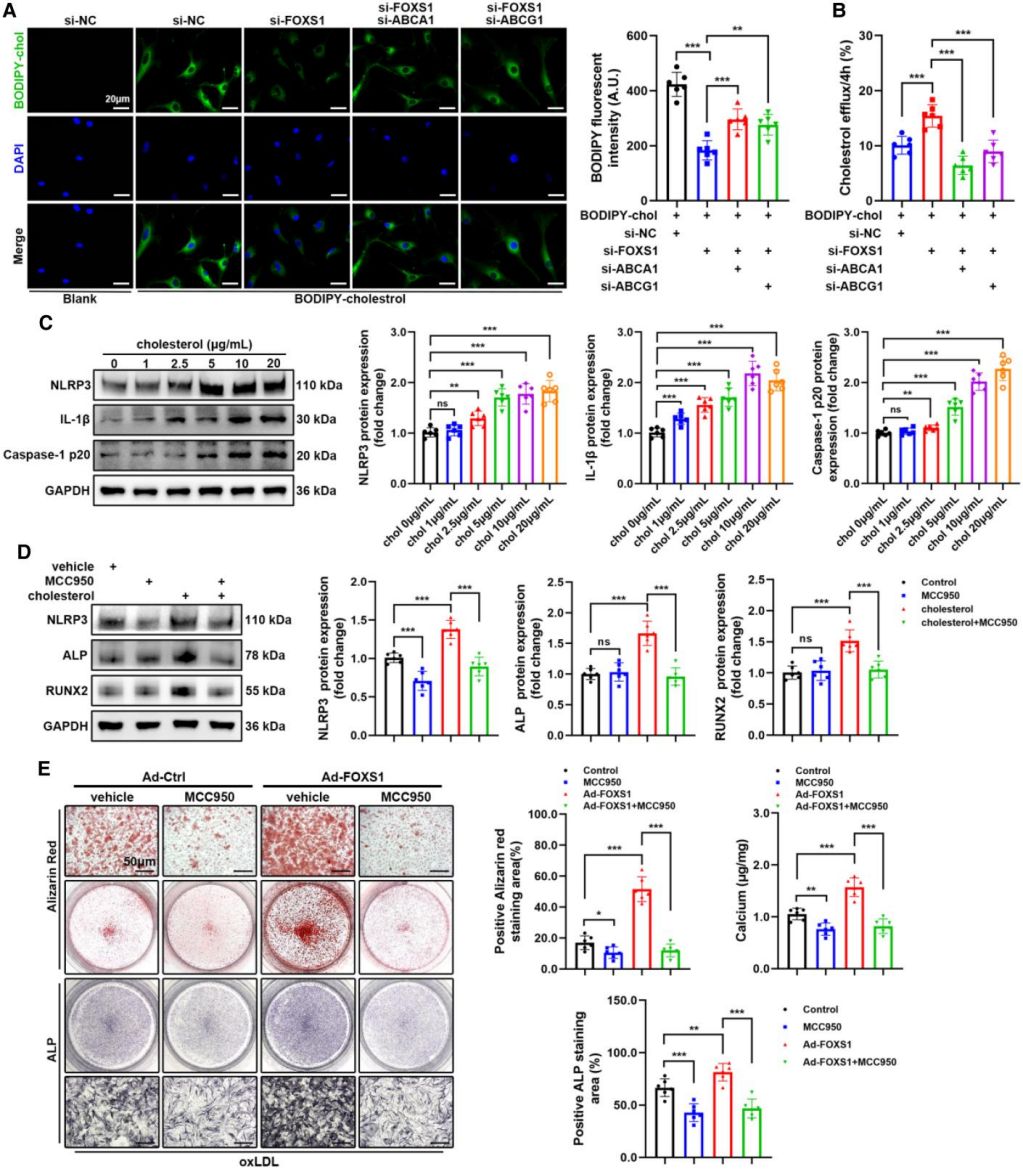
Cholesterol transport dysfunction drives NLRP3 inflammasome activation and osteogenic differentiation in VICs. (*A* and *B*) VICs were incubated with Bodipy-cholesterol (1 μM) under si-FOXS1, si-ABCA1, and si-ABCG1 for 4 h. Representative images of Bodipy-cholesterol-labelled VICs are shown. And cholesterol efflux of VICs was evaluated (*B*). Scale bar = 20 μm; *n* = 6. (*C*) Western blot analysis of protein levels in VICs treated with cholesterol in different concentrations (*n* = 6). (*D*) Western blot analysis of protein levels in VICs treated with cholesterol (10 μg/mL) and MCC950 (10 μM) (*n* = 6). (*E*) Alizarin Red staining, calcium content, and ALP staining of VICs treated with Ad-FOXS1 and MCC950 (10 μM) (*n* = 6). Scale bar = 50 μm. Values are the mean ± SD. *P* values were calculated using one-way ANOVA followed by Bonferroni multiple comparisons test. **P < 0.01, ***P < 0.001.

To test our hypothesis, VICs were treated with oxLDL for various durations, and then we found that the cholesterol levels in VICs increased over time, reaching a high level after 4 h (see Supplementary material online, *Figure S9*). To more directly observe the regulatory effect of FOXS1 on cholesterol transport in VICs, we treated VICs with Bodipy-cholesterol for various durations. Immunofluorescence results showed that the Bodipy-cholesterol fluorescence signal in VICs gradually increased (see Supplementary material online, *Figure S9*). Subsequent treatment of VICs with Bodipy-cholesterol followed by silencing FOXS1, ABCA1, or ABCG1 showed that silencing FOXS1 reduced intracellular cholesterol fluorescence signals and increased cholesterol efflux efficiency, while silencing ABCA1 or ABCG1 reversed these effects (*Figure 5A* and *B*). Additionally, Oil Red O staining showed that lipid deposition in the aortic valves of Apoe^−/−^Foxs1^−/−^ mice was significantly reduced, but there were no significant changes in the aortic root (see Supplementary material online, *Figure S10*). These results indicate that FOXS1 affects cholesterol transport in VICs through ABCA1 and ABCG1, regulating intracellular cholesterol levels. Moreover, ABCA1 seems to have a greater impact than ABCG1, which was consistent with the results above.

To explore the role of cholesterol in the inflammasome activation of VICs, different concentrations of cholesterol were used to treat VICs. Western blot results showed that NLRP3, IL-1β, and caspase-1 p20 levels increased with concentration (*Figure 5C*). This result is consistent with that observed in human and mouse aortic valves (see Supplementary material online, *Figure S11*), which confirms our previous conjecture that elevated LDL cholesterol levels could promote inflammasome activation in VICs. Besides，we confirmed that FOXS1 silencing could inhibit the inflammasome activation induced by oxLDL (see Supplementary material online, *Figure S12*). Moreover, silencing ABCA1 or ABCG1 reversed the inflammasome inhibition caused by silencing FOXS1 (see Supplementary material online, *Figure S13*). These results suggested that oxLDL mediates cholesterol accumulation through FOXS1 to promote inflammasome activation. To investigate the effect of inflammasome on osteogenic differentiation in VICs, we treated VICs with cholesterol and the NLRP3 inhibitor MCC950, and then we found that MCC950 significantly inhibited the up-regulation of NLRP3, ALP, and RUNX2 induced by cholesterol (*Figure 5D*). Subsequent Alizarin Red and ALP staining confirmed that MCC950 reduced the increase of calcium salt deposition and ALP level of VICs induced by the overexpression of FOXS1 (*Figure 5E*). These results indicate that FOXS1 promotes osteogenic differentiation in VICs through ABC transport-mediated cholesterol accumulation and NLRP3 inflammasome activation.

### 3.6 IMM-H007 inhibits oxLDL-induced osteogenic differentiation of VICs by increasing ABCA1 levels

**Figure 6:**
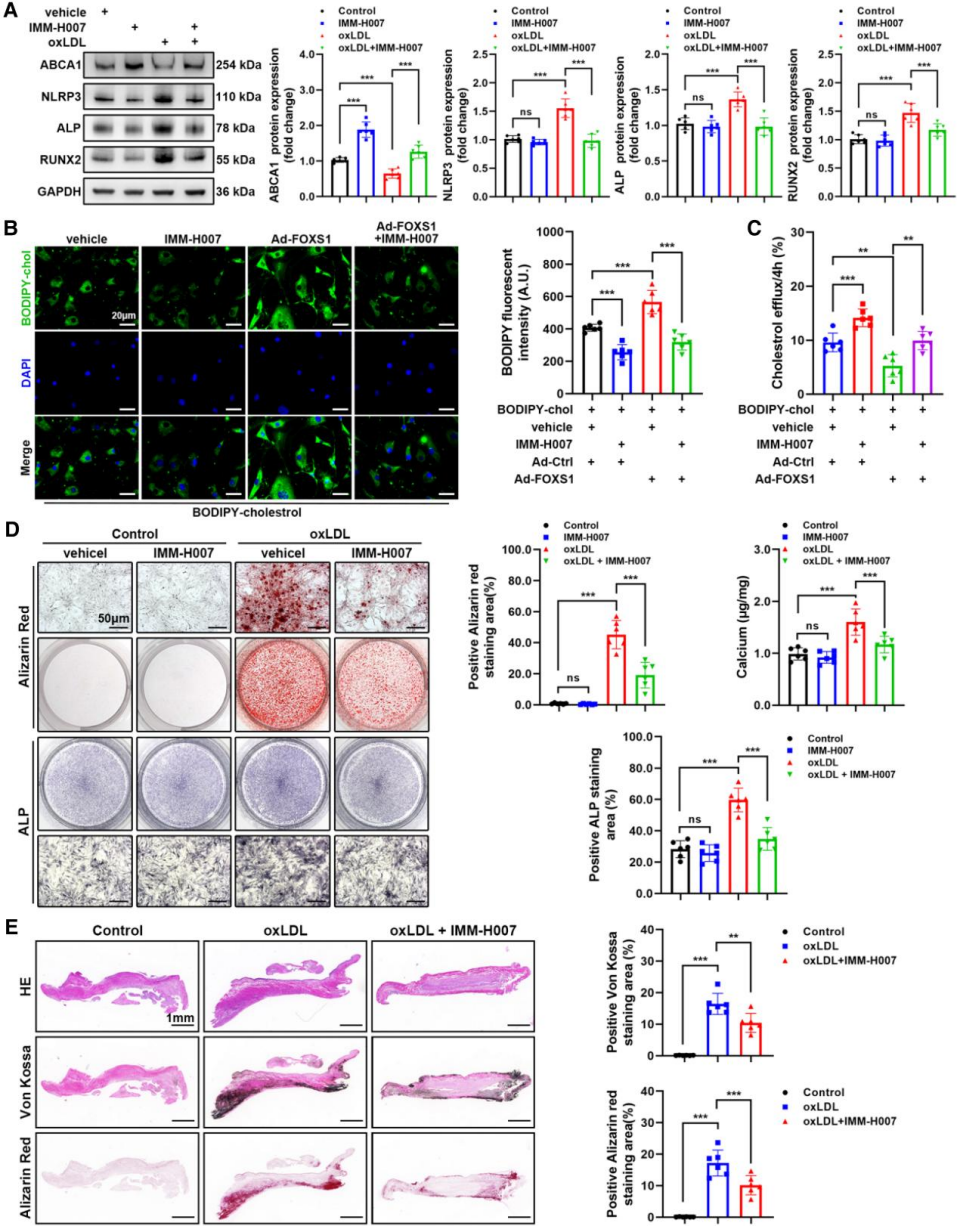
IMM-H007 inhibits oxLDL-induced osteogenic differentiation of VICs by increasing ABCA1 levels. (*A*) Western blot analysis of protein levels in VICs treated with IMM-H007 (10 μM) and oxLDL (*n* = 6). (*B* and *C*) Representative images of Bodipy-cholesterol-labelled VICs treated with Ad-FOXS1 and IMM-H007 (10 μM). And cholesterol efflux of VICs was evaluated (*C*) (*n* = 6). Scale bar = 20 μm. (*D*) Alizarin Red staining, calcium content, and ALP staining of VICs treated with oxLDL and IMM-H007 (10 μM) (*n* = 6). Scale bar = 50 μm. (*E*) HE, Von Kossa, and Alizarin Red staining of aortic valves treated with IMM-H007 (10 μM) and oxLDL for 21 days *in vitro* (*n* = 6). Scale bar = 1 mm. Values are the mean ± SD. *P* values were calculated using one-way ANOVA followed by Bonferroni multiple comparisons test. **P < 0.01, ***P < 0.001.

IMM-H007 is an inhibitor of ABCA1 degradation.^41^ To explore the potential of ABCA1 as a therapeutic target, VICs were treated with IMM-H007 for various durations. Western blot analysis showed that ABCA1 increased with the treatment duration of IMM-H007 (see Supplementary material online, *Figure S14*). Moreover, IMM-H007 reversed the down-regulation of ABCA1 and the up-regulation of NLRP3, ALP, and RUNX2 caused by oxLDL (*Figure 6A*). Besides, IMM-H007 reversed the cholesterol efflux impairment caused by overexpression of FOXS1, promoting cholesterol outflow and reducing intracellular cholesterol levels (*Figure 6B* and *C*). IMM-H007 also reversed the increase of calcium salt deposition and ALP level in VICs induced by oxLDL (*Figure 6D*). The efficacy of IMM-H007 in reducing valve calcification induced by oxLDL was further validated in an *ex vivo* osteogenic differentiation model (*Figure 6E*).

### 3.7 FOXS1 regulated ABCA1 and ABCG1 by inhibiting the BSCL2-mediated PPARγ/LXRα axis

**Figure 7:**
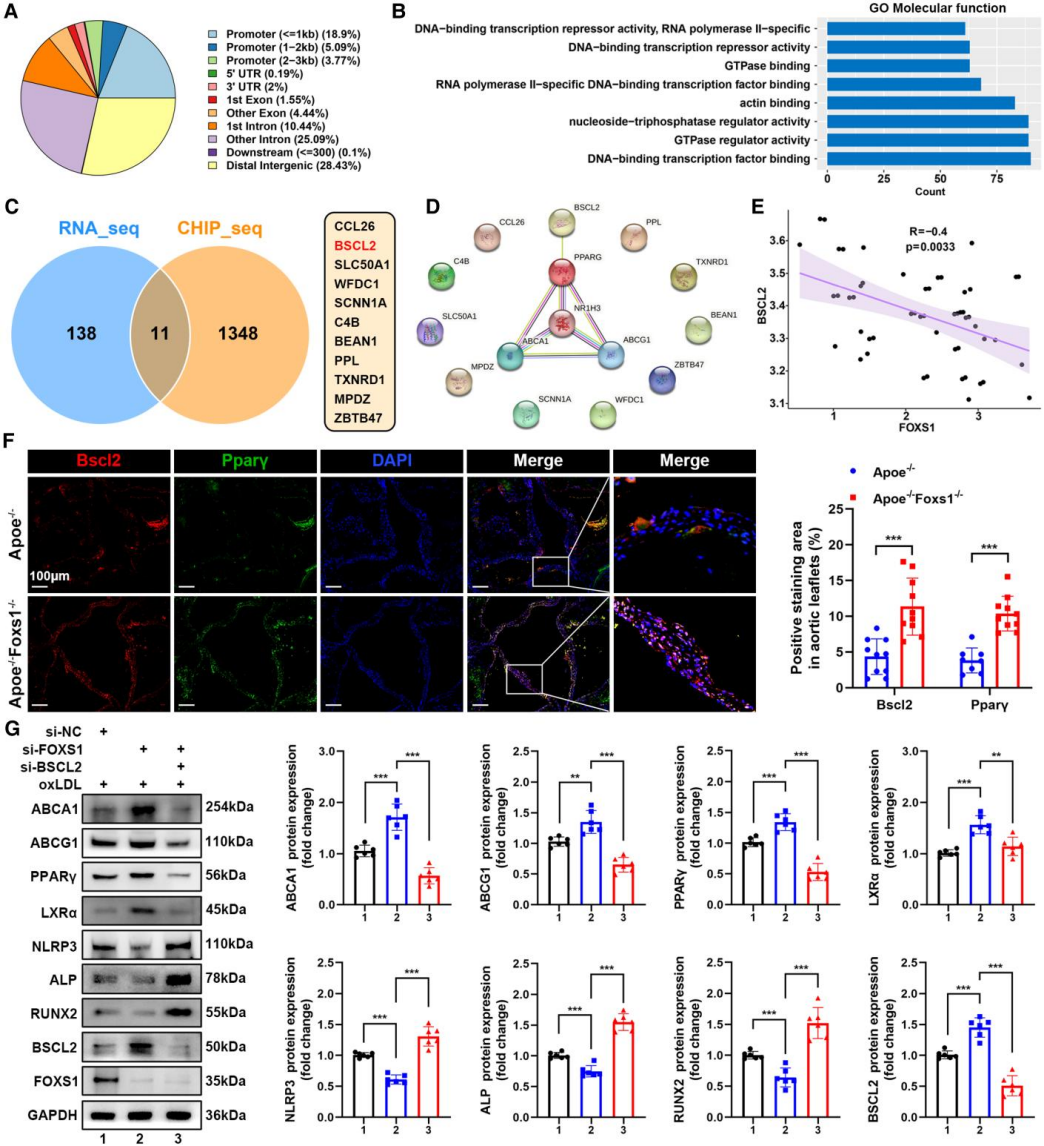
FOXS1 regulated ABCA1 and ABCG1 by inhibiting the BSCL2-mediated PPARγ/LXRα axis. (*A*) Pie chart showing the distribution of FOXS1 peaks in different genomic regions as indicated in ChIP-seq of VICs. (*B*) GO molecular function enrichment analysis of FOXS1 peaks. (*C* and *D*) Venn diagram interaction of promoters in ChIP-seq and DEGs in RNA-seq. The interaction between the 11 common genes and PPARγ, LXRα (NR1H3), ABCA1, and ABCG1 are analysed (*D*) using the STRING. (*E*) Correlation analysis between FOXS1 and BSCL2 in human aortic valves. (*F*) Immunofluorescence staining of Bscl2 and Pparγ in aortic valves from Apoe^−/−^ and Apoe^−/−^Foxs1^−/−^ mice fed with HFD (*n* = 10). Scale bar = 100 μm. DAPI was used for nuclear counterstaining. (*G*) Western blot analysis of protein levels in VICs treated with si-FOXS1 and si-BSCL2 (*n* = 6). Values are the mean ± SD. *P* values were calculated using two-tailed Pearson correlation analysis (*E*), unpaired two-tailed Student’s *t*-test (*F*), or one-way ANOVA followed by Bonferroni multiple comparisons test (*G*). **P < 0.01, ***P < 0.001.

As a TF, FOXS1 has the ability to bind to DNA and regulate gene expression. However, the specifics of FOXS1’s transcriptional regulation function are not yet understood. To explore the potential downstream targets of FOXS1 and explain why FOXS1 regulate ABCA1 and ABCG1 expression, we conducted ChIP-seq targeting FOXS1 in VICs. Overall, FOXS1-bound sites included promoters (27.76%), distal intergenic (28.43%), intron (35.53%), and exons (5.99%; *Figure 7A*). The FOXS1 peak-related genes significantly enriched in the DNA-binding transcription repressor activity (*Figure 7B*).

In order to obtain gene targets that are directly related to FOXS1 in transcriptional regulation, we identified the genes that positively (number = 81) or negatively (number = 68) regulated by FOXS1 in RNA-seq (see Supplementary material online, *Figure S15*). These 149 DEGs were interacted with the genes of which promoter bound by FOXS1 in ChIP-seq. Then 11 common genes were identified (*Figure 7C*. Using the Search Tool for the Retrieval of Interacting Genes (STRING) database, we found that only Berardinelli–Seip congenital lipodystrophy 2 (BSCL2) might be associated with ABC transport proteins through PPARγ and LXRα (*Figure 7D*). Subsequently, we found a significant negative correlation between BSCL2 and FOXS1 gene expression in Bulk RNA-seq mentioned above (*Figure 7E*). Furthermore, in Foxs1^−/−^ mice, Bscl2 and Pparγ were significantly up-regulated in the aortic valves as evidenced by immunofluorescence staining (*Figure 7F*). Then we performed DNA sequence alignment to identify the BSCL2 promoter sites that FOXS1 might bind (see Supplementary material online, *Figure S16A*) and carried out site-directed mutagenesis on the binding region, which is near the transcription start site (TSS). qRT-PCR confirmed the regulatory relationship between FOXS1 and BSCL2 (see Supplementary material online, *Figure S16B*). A dual-luciferase reporter assay confirmed the binding of FOXS1 to the BSCL2 promoter (see Supplementary material online, *Figure S16C*). Western blot analysis indicated that silencing BSCL2 significantly reversed the increase in PPARγ, LXRα, ABCA1, and ABCG1 and the decrease in NLRP3, ALP, and RUNX2 caused by FOXS1 silencing (*Figure 7G*). Meanwhile, we found that silencing BSCL2 significantly reversed the increased cholesterol efflux efficiency caused by silencing FOXS1 (see Supplementary material online, *Figure S17*), as well as the reduction in calcium salt deposition and the decrease in ALP level (see Supplementary material online, *Figure S18*). Additionally, the anti-calcification effect of BSCL2 was confirmed in an *ex vivo* osteogenic differentiation model (see Supplementary material online, *Figure S19*).

### 3.8 BSCL2 mitigates aortic valve calcification *in vivo*

To explore the therapeutic effect of BSCL2 on CAVD *in vivo*, we administered HFD-fed Apoe^−/−^ mice with vehicle or recombinant murine Bscl2 (rmBscl2) for 24 weeks (see Supplementary material online, *Table S4*). rmBscl2 ameliorated aortic valve calcification in Apoe^−/−^ mice, as evidenced by the echocardiography (see Supplementary material online, *Figure S20A* and *S20B*), Von Kossa, Alizarin Red, Masson, and Oil Red O staining (see Supplementary material online, *Figure S20C*). Additionally, immunofluorescence staining showed that rmBscl2 increased the expression of PPARγ and LXRα in the aortic valve (see Supplementary material online, *Figure S21*). Together, these results prove that BSCL2 could mitigate calcification in the aortic valve, potentially becoming a new therapeutic target for CAVD.

## 4. Discussion

CAVD is a complex multifactorial disease, the pathomechanisms of which have not yet been fully elucidated.^42^ At present, there is a lack of pharmacological interventions proven to effectively impede or decelerate the advancement of CAVD. As a result, investigating the initial pathogenesis of CAVD and pinpointing novel therapeutic targets holds significant importance. Lipid deposition is considered to be an early biomarker of CAVD,^4^ in which oxLDL mediates chronic inflammation and accelerates valve calcification, but the mechanism remains unclear. This study is the first to report on FOXS1, a TF activated by oxLDL in VICs, which promotes the osteogenic differentiation of VICs and the progression of CAVD by mediating cholesterol transport disruption and inflammatory activation. FOXS1 inhibits gene transcription by binding to the promoter region of BSCL2, thereby suppressing the PPARγ–LXRα–ABC transporter axis, leading to obstructed cholesterol efflux, cholesterol accumulation in VICs, activation of the NLRP3 inflammasome, and promotion of osteogenic differentiation of VICs, which ultimately exacerbates valvular calcification (*Graphical abstract*). Overall, our findings identified a novel regulatory pathway from lipid deposition to osteogenic differentiation, contributing to the pathogenesis of CAVD.

Initially, up-regulated TFs in CAVD were identified through bulk RNA-seq analysis, with FOXS1 being pinpointed as a potential key player in oxLDL-induced osteogenic differentiation of VICs via qRT-PCR. Subsequent validation using an *in vitro* VIC osteogenic differentiation model confirmed the regulatory role of FOXS1 in oxLDL-mediated osteogenic differentiation. Further investigation through RNA-seq analysis revealed that FOXS1 can modulate cholesterol transport in VICs by regulating the expression of ABC transporters ABCA1 and ABCG1. Previous studies have indicated that ABCA1 and ABCG1 facilitate the efflux of cholesterol from macrophages, a mechanism known as RCT, thereby mitigating intracellular cholesterol buildup and inhibiting foam cell formation.^31–34^ This process has been shown to have a substantial impact on the reduction of vascular diseases, including atherosclerosis. Individuals with a deficiency in the ABCA1 gene are predisposed to atherosclerosis, as evidenced by the manifestation of Tangier disease.^36^ Nonetheless, the involvement of ABC transporters and cholesterol accumulation in VICs had not been previously explored, thereby introducing a novel perspective on CAVD. In order to comprehensively examine the validity of these theories in VICs, we employed Bodipy-cholesterol to visually illustrate the ABC transporter-mediated cholesterol release regulated by FOXS1. Furthermore, we established that heightened intracellular cholesterol accumulation can trigger the activation of the NLRP3 inflammasome, facilitate the osteogenic differentiation of VICs, and ultimately result in calcification. These findings offer novel perspectives on the involvement of lipid deposition in the pathogenesis of CAVD.

As a constituent of the FOX family, FOXS1 has received limited attention in research. The potential regulatory role of FOXS1 on ABC transport proteins may be linked to its TF function. To investigate this, we performed ChIP-seq analysis and compared the results with RNA-seq data to identify potential downstream target genes directly regulated by FOXS1 binding to promoters. Despite not identifying any genes associated with ABCA1 or ABCG1 in the target gene list, we hypothesize that FOXS1 may regulate both genes through control of a common upstream gene. PPARγ or LXRα, serving as shared upstream targets of ABCA1 and ABCG1, may act as the intermediary connection between FOXS1 and ABC transporters.^43,44^ Utilizing the STRING database for analysis, we found that BSCL2 may act as a downstream target of FOXS1 and be involved in the regulation of PPARγ, albeit the precise mechanism remains undetermined.

The BSCL2 gene encodes the protein seipin, which plays a crucial role in lipid formation and homeostasis.^45,46^ Deletion of the BSCL2 gene has been linked to the development of human congenital generalized lipodystrophy and cardiac dysfunction.^47–49^ However, the potential involvement of BSCL2 in CAVD has not been reported. Previous studies have implicated PPARγ in BSCL2-related cognitive impairments,^50,51^ and PPARγ has been identified as a potential therapeutic target for CAVD.^52^ These findings suggest that the regulatory influence of BSCL2 on PPARγ may be critical in the FOXS1-mediated regulation of ABC transporters. Subsequent experiments validated these hypotheses, elucidating the regulatory role of FOXS1, in conjunction with BSCL2, in modulating the PPARγ–LXRα–ABC transporter signalling pathway and promoting cholesterol transport dysfunction and NLRP3 inflammasome activation. Recent studies have demonstrated that NLRP3 activation induces the expression of osteogenic markers in VICs such as RUNX2, ALP, and osteocalcin via the IL-1β and IL-18 secretion and the NF-κB and MAPK signalling pathways. These findings suggest that NLRP3-mediated inflammation creates a pro-osteogenic microenvironment, driving VICs towards calcification, which is consistent with our findings.^53,54^ The results of this investigation shed light on the processes through which initial lipid accumulation contributes to persistent inflammation, underscoring its potential importance in the prevention and early treatment of CAVD.

The present study has several limitations. First, the precise mechanism by which oxLDL induces the up-regulation of FOXS1 remains unclear. The role and underlying mechanisms of FOXS1 in human diseases are currently underexplored in the literature. Further research is needed to elucidate the relationship between oxLDL and FOXS1 expression in order to better understand their potential implications in disease pathogenesis. Secondly, oxysterols, as part of oxLDL, have the effect of activating LXR to inhibit inflammation, which is in contrast to the pro-inflammatory effect of oxLDL in the present study but was not investigated here. As is well known, oxLDL has the ability to activate inflammation, contributing significantly to the inflammatory environment in diseases such as atherosclerosis.^55^ Oxysterols, as components of oxLDL, can activate LXR and regulate cholesterol homeostasis as well as suppress inflammation, which is in contrast to the effects of oxLDL.^56^ In our study, oxLDL inhibited the expression of LXRα and led to cholesterol transport disorders and inflammation activation by up-regulating a series of pathways including FOXS1, indicating that oxysterols failed to exert their anti-inflammatory effects or its effect was too weak. The reason may be that oxLDL suppressed the abundant expression of LXR. Oxysterols could not reverse the pro-inflammatory effects of oxLDL by activating LXR with low expression levels. This may also be the reason why oxLDL tends to exert pro-inflammatory effects rather than the anti-inflammatory effects of oxysterols.^57^ However, whether oxysterols have anti-inflammatory effects in VICs and whether their relationship with oxLDL is as hypothesized need further study. Thirdly, the therapeutic compound IMM-H007, which targets ABCA1, lacks empirical support from *in vivo* experiments. While IMM-H007 has demonstrated efficacy in reducing circulating LDL levels by inhibiting ABCA1 degradation in the liver and macrophages, thus potentially mitigating atherosclerosis, its specific therapeutic mechanism in relation to VICs remains uncertain. To address this gap, further research is needed to develop stable and effective targeted drug delivery techniques for investigating the direct therapeutic effects of IMM-H007 on the aortic valve. Fourthly, the *ex vivo* osteogenic differentiation model was chosen for several reasons. This model provides a controlled environment that preserves the native extracellular matrix and cell–cell interactions. It also simplifies the complexities of *in vivo* experiments by eliminating systemic factors, allowing for a more focused examination of specific biological or mechanical factors. This model is particularly useful for proof-of-principle studies, as it offers a balance between *in vitro* and *in vivo* experimentation while also addressing ethical considerations related to animal testing. However, this model has significant limitations when used *in vivo*. The absence of systemic factors and the natural biological environment can lead to results that may not fully translate to clinical settings. Fifthly, the western diet-fed Apoe^−/−^ mice model has inherent limitations. Numerous alternative mouse models for CAVD have been established, necessitating further validation of mechanisms in various animal models. Sixthly, the globally knockout of FOXS1 for the study of CAVD has certain limitations. Although our results show that FOXS1 is significantly up-regulated only in VICs of calcified valves compared to normal valves, rather than in other types of cells, we are not clear whether FOXS1 affects the functions of other organs or cells such as macrophages and endothelial cells, and whether it contributes to CAVD. In the future, it may be necessary to utilize new targeted technologies for more precise gene regulation and to further comprehensively explore its mechanisms. Seventhly, While our findings demonstrate that rmBscl2 attenuates aortic valve calcification in Apoe^−/−^mice, several critical issues must be resolved prior to clinical translation. Substantial refinement of the delivery route is required. Although intraperitoneal injection achieved detectable bioavailability in our murine model, alternative strategies such as nanoparticle-mediated targeted delivery or tissue-penetrating peptide conjugates warrant exploration to enhance therapeutic efficacy while minimizing invasiveness. Besides, comprehensive safety profiling remains imperative. While no overt adverse effects (e.g. weight loss, organ toxicity, or behavioral abnormalities) were observed in our study, the long-term systemic consequences of rmBscl2 administration, particularly its immunogenicity and off-target effects, remain uncharacterized. Future investigations should incorporate multi-organ histopathological analyses, cytokine profiling, and anti-drug antibody assays to establish a therapeutic safety window. These translational challenges underscore that our current work represents a preclinical proof of concept rather than a directly actionable therapy. Nevertheless, the oxidation-dependent activation of FOXS1 identified herein provides a mechanistic framework for developing next-generation calcification inhibitors with enhanced clinical applicability. Finally, this study does not include data regarding the impact of FOXS1, BSCL2, or other potential targets in the treatment of early-stage CAVD patients. In this study, PPARγ-/LXRα-related treatment strategies, such as rosiglitazone, pioglitazone, and lanifibranor, have shown therapeutic potential in diabetes and atherosclerosis. ABC transporter-related strategies such as IMB2026791 and R3R-01 are also in clinical trials for atherosclerosis. These strategies may be beneficial for CAVD in the future. However, there are few existing studies on FOXS1 and BSCL2, and targeted therapies are limited. In the future, new precise treatment methods may be developed through drug screening or gene-editing therapies, as well as conducting long-term clinical cohort studies to address these gaps in knowledge.

Translational perspective Chronic inflammation triggered by lipid deposition is a pivotal driver of CAVD. Thus, uncovering the molecular mechanisms of LDL-induced chronic inflammatory activation in VICs is crucial for disease prevention and treatment. This study shows that TFs FOXS1 and the BSCL2/PPARγ/LXRα axis are key regulators of lipid deposition during early-stage valve calcification. They affect cholesterol transport and inflammatory activation in VICs, accelerating CAVD progression. Therapeutic strategies targeting PPARγ, LXRα, and ABC transporters have shown promise in treating atherosclerotic and metabolic chronic diseases such as diabetes and may also benefit CAVD patients. However, research on FOXS1 and BSCL2 is currently limited, and targeted therapies are scarce. Additionally, the safety of targeting FOXS1 and BSCL2 in clinical settings requires further investigation, as potential side effects and long-term impacts are not yet fully understood. Developing new precise treatments through drug screening or gene editing could be a promising direction for future research.

## 5. Conclusion

In conclusion, our current work demonstrates that FOXS1 promotes the dysfunction of cholesterol transport and the activation of NLRP3 inflammasome, thereby enhancing the osteogenic differentiation of VICs in CAVD. We have shown that as a TF, FOXS1 can bind to the promoter of BSCL2, inhibit its transcriptional expression, and thereby suppress ABC transporters via the PPARγ–LXRα axis. This leads to impaired cholesterol efflux, resulting in intracellular cholesterol accumulation, inflammasome activation, and ultimately accelerating valvular calcification. These findings may offer potential therapeutic strategies for the early prevention and treatment of CAVD patients in the future.

## Supplementary Material

cvaf159_Supplementary_Data

## Data Availability

The bulk RNA-seq data and scRNA-seq data were obtained from the GEO database (GSE148219, GSE76718, GSE153555, and GSE180278). The ChIP-seq data have been uploaded and available on the SRA database (PRJNA1126021). The data, methods used in the analysis, and materials will be made available to any researcher for purposes of reproducing the results or replicating the procedure on reasonable request to the corresponding author.
