# Supplementary material for: Oxidized LDL-induced FOXS1 mediates cholesterol transport dysfunction and inflammasome activation to drive aortic valve calcification
Source: Cardiovasc Res. 2025 Sep 24;121(12):1941–55. doi: 10.1093/cvr/cvaf159 (PMC12551391; doi:10.1093/cvr/cvaf159)
Supplement: cvaf159_Supplementary_Data [file cvaf159_supplementary_data.zip › Revised Supplemental Material-CardiovasRes 20250322.docx]

**SUPPLEMENTAL MATERIAL**

**oxLDL-induced FOXS1 mediates cholesterol transport dysfunction and inflammasome activation to drive aortic valve calcification**

1. **Supplementary methods**

**1.1 Human samples and ethics**

Aortic valves that had not calcified were sourced from individuals undergoing cardiac transplantation due to dilated cardiomyopathy. In contrast, calcified aortic valves were harvested from patients who were candidates for aortic valve replacement surgery specifically due to CAVD. This research was conducted in strict accordance with the ethical guidelines set forth in the Declaration of Helsinki and was granted approval by the Systematic Review Committee of Tongji Medical College, which is affiliated with Huazhong University of Science and Technology. Before any surgical procedures were performed, we ensured that all participants provided their written, informed consent.

**1.2 Animal study approval**

All procedures involving animals were in strict compliance with the "Guide for the Care and Use of Laboratory Animals," as published by the US National Institutes of Health (NIH Publication No. 85-23, revised 1996). The Animal Care and Use Committee at Tongji Medical College provided the necessary approval for these procedures. Apoe^-/-^ and Apoe^-/-^Foxs1^-/-^ mice were purchased from GemPharmatech Co., Ltd.

**1.3 Bulk RNA sequencing analysis**

The bulk RNA sequencing (RNA-seq) raw data of aortic valves were sourced from the European Nucleotide Archive and their corresponding information can be accessed on the Gene Expression Omnibus (GEO) database (GSE148219, GSE76718, GSE153555). Dataset GSE148219 includes 8 control and 7 calcified aortic valve samples. Dataset GSE76717 includes 8 normal and 9 calcified aortic valve samples. Dataset GSE153555 includes 10 normal and 10 calcified aortic valve samples. The raw data underwent quality control using Fastqc 0.11.9, mapping with Hisat2 v2.2.1, and quantification with featureCounts v2.0.1 on a Linux platform to generate the count matrix. Subsequently, the matrix was filtered based on counts per million (cpm) > 0 using edgeR, followed by normalization and identification of differentially expressed genes (DEGs) using DESeq2 on R 4.2.1.

**1.4 List of transcription factors**

The list of human transcription factors (TFs) is available from SCENIC’s GitHub repository. (https://github.com/aertslab/pySCENIC/tree/master/resources).

**1.5 Single-cell RNA sequencing analysis**

Single-cell RNA-seq raw data was retrieved from the Gene Expression Omnibus database (GSE180278) for the purpose of reanalysis.^33^ The hyperlipidemia model consisted of male mice with Ldlr or Apoe knocked out and fed a western diet for 8-10 weeks, while the control group comprised wild type C57BL/6J mice fed with a chow diet.

**1.6 Cell culture**

Primary human aortic VICs were isolated from non-calcified aortic valves as described previously.^34,35^ Following the triple washing of the aortic valves in phosphate-buffered saline (PBS), the valves were subjected to digestion in 1 mg/mL type I collagenase for a duration of 12 hours at 37 ℃ in an environment containing 5% CO2. Subsequently, the resulting cell suspension underwent a gentle centrifugation at 1000 rpm for 10 minutes, following which the resuspended and isolated primary VICs were cultivated in high glucose Dulbecco’s modified Eagle’s medium (DMEM, Gibco) supplemented with 10% fetal bovine serum (Gibco) under conditions of 5% CO2 at 37°C. The third passage VICs from the subcultures were used for cell experiments. Without any other specifications, oxLDL was subjected to various treatments for distinct periods depending on the assay: Western blot analysis was performed after 3 days, ALP staining after 7 days, Alizarin red staining after 21 days, and the ex-vivo valve osteogenic differentiation model was assessed over a period of 2 months. The oxLDL was replenished every 3 days. All experiments were conducted on VICs from distinct batches, with n values denoting separate experiments.

**1.7 Ex-vivo valves osteogenic differentiation model**

Aortic valve leaflets from patients undergoing cardiac transplantation were harvested and sectioned into approximately 2 mm * 2 mm fragments, then cultured in 12-well plates with DMEM supplemented with oxLDL, IMM-H007 or rhBSCL2 for 2 months. Then these samples were washed thrice in PBS and fixed in 4% paraformaldehyde, followed by HE, Von Kossa and Alizarin Red staining to assess calcium salt deposition.

**1.8 Quantitative real time polymerase chain reaction (qRT-PCR)**

Total RNA was extracted from VICs using the Total RNA Isolation Kit (Vazyme, RC112-01), and cDNA was synthesized using the HiScript III RT SuperMix (Vazyme, R323-01), both in accordance with the manufacturer's instructions. For quantitative real-time PCR (qRT-PCR), reactions were set up in optical 96-well plates with SYBR qPCR Master Mix (Vazyme, Q711-02) and carried out on a Step One Real-Time PCR System (Applied Biosystems, Foster City, CA, USA), following the protocol provided by the manufacturer. GAPDH was used as an endogenous control for normalization. Relative gene expression levels were determined using the ΔΔCt method, with the primer sequences listed in Table S2.

**1.9 Western blot**

Protein extraction was performed using radioimmunoprecipitation assay (RIPA) buffer (New Cell & Molecular Biotech, WB3100), enriched with a cocktail of protease and phosphatase inhibitors (New Cell & Molecular Biotech, P002). Protein concentrations in the lysates were measured employing the bicinchoninic acid (BCA) protein assay kit (Beyotime Biotechnology, P0011). For western blot analysis, equal quantities of protein from each sample were resolved on 4%-12% sodium dodecyl sulfate polyacrylamide gels (ACE Biotechnology, ET15412Gel). After electrophoresis, proteins were transferred to polyvinylidene fluoride (PVDF) membranes (Millipore, 03010040001) and incubated with a blocking solution of 5% nonfat dry milk in Tris-buffered saline containing 0.5% Triton X-100 (TBS-T). Membranes were then incubated with specific primary antibodies overnight at 4°C, followed by a 1-hour incubation with appropriate secondary antibodies. Bands were visualized using an enhanced chemiluminescence detection system (New Cell & Molecular Biotech, P002), and their intensities were quantified with Image J software version 1.8 (National Institutes of Health).

**1.10 Immunohisochemistry staining of aortic valves**

After the process of antigen retrieval, paraffin-embedded sections of aortic valve tissues were incubated with the primary antibody overnight at 4°C to allow for optimal antibody binding. This was followed by incubation with a horseradish peroxidase (HRP)-conjugated secondary antibody to amplify the signal.

The immunofluorescent images were then captured using a high-resolution fluorescence microscope from Carl Zeiss (Jena, Germany), which provided clear and detailed visualization of the antigen-antibody complexes. These images were further processed and merged using Image J software version 1.8 (National Institutes of Health) to create composite figures that showcased the staining patterns and intensities across different samples.

**1.11 Immunofluorescence staining**

Aortic valve frozen sections were air-dried for 20 minutes, fixed with 4% paraformaldehyde for 30 minutes, and permeabilized with 0.1% Triton X-100 in PBS for 15 minutes. The sections were incubated with a primary antibody, followed by a fluorescently conjugated secondary antibody, and counterstained with DAPI to visualize cell nuclei.

**1.12 Adenovirus-mediated overexpression**

Recombinant adenovirus vectors containing the specified gene were procured from WZ Biosciences Inc. (Shandong, China). VICs were seeded at a density of 1.0 × 10^5^ cells per well in 6-well plates and exposed to adenovirus at a multiplicity of infection (MOI) of 25 (refer to Figure S3 for details on MOI value screening). Following a 48-hour incubation period with the adenovirus, VICs were utilized for subsequent experimental interventions.

**1.13 Small interfering RNA-mediated silencing**

siRNAs aimed at FOXS1, ABCA1, ABCG1, BSCL2, and a non-targeting negative control siRNA (si-NC) were custom-designed and synthesized by RiboBio in Guangzhou, China. The specific sequences of these siRNAs are detailed in Table S2. Prior to transfection, VICs were seeded onto 6-well plates and allowed to adhere for 24 hours. Transfection was carried out using Lipofectamine 3000 (Thermo Fisher Scientific, L3000075), following the protocol provided by the manufacturer. After transfection, the cells were cultured for an additional 48 hours to allow for siRNA-mediated gene silencing before proceeding with subsequent experimental analyses.

**1.14 Experimental animals and protocols**

*Apoe^-/-^* mice and Foxs1^-/-^ mice were purchased from GemPharmatech (Nanjing, China). Based on the Foxs1 gene structure, exon1 of the Foxs1-201 (ENSMUST00000099200.3) transcript was identified as the optimal target for gene knockout, as it encompasses the entire coding sequence. Disrupting this region would lead to a loss of protein function. In our study, we employed CRISPR-Cas9 technology to target the Foxs1 gene. The process involved microinjecting the CRISPR-Cas9 system into fertilized eggs of T001458-Apoe mice. These fertilized eggs were then implanted to yield F0 mice that tested positive for the desired modification, as confirmed by PCR and sequencing. Finally, a stable F1 generation mouse model was established by crossing the positive F0 mice with T001458-Apoe mice. To generate the Foxs1 knockout mice in Apoe^-/-^ background, the Foxs1^-/-^ mice were crossed with Apoe^-/-^ mice, which finally yielded Apoe^-/-^Foxs1^-/-^ mice. Tail snips were used to collect genomic DNA from the progeny, which was then identified by PCR using certain primers and direct sequencing. The following primer sequences were used to amplify the genomic DNA fragments containing the gRNA region end caps (F1-R1): forward: 5’-CTTCTCACAGTTCCATATGGCAGATAC-3’; reverse: 5’-CACGCACACACACGAATGTAATACA-3’ or containing the complete knockout region (F2-R2): forward: 5’-TTCGGAATGAGTCCTCAGACTACCA-3’; reverse: 5’-CTTACCCTGGAAGAAGCAGCAAAT-3’. The F2-R2 PCR products were further sequenced to verify that the targeted region was correctly deleted. Littermate Apoe^-/-^Foxs1^+/+^ mice (Apoe^-/-^ mice for short) were used as controls.

Eight-week-old male *Apoe^-/-^* (n=10) and Apoe^-/-^Foxs1^-/-^ (n=10) mice were fed with western diet for 24 weeks. For studies of BSCL2, eight-week-old male Apoe^-/-^ mice were randomly allocated to groups: (1) mice fed with western diet and normal saline solution; (2) mice fed with western diet supplemented with rmBscl2 (10mg/kg) for 24 weeks. The recombinant protein or normal saline solution was administered by intraperitoneal injection twice weekly.The mice were maintained in a sterile, temperature-regulated facility with a 12-hour alternating light and dark cycle. Allocation concealment with sequentially numbered method was used to eliminate selection bias in intervention assignment. Blinding procedures with regard to masking of group/treatment assignment were used to ensure the experimenter unaware of the treatment allocation.

Following a 24-week regimen of a western-style diet, the hemodynamic parameters of the mice were assessed using transthoracic echocardiography with an 18-38 MHz phased-array probe (MS400) linked to a Vevo 2100 Imaging system, under anesthesia induced by 2.5% isoflurane. Subsequently, the mice were humanely euthanized via intravenous administration of a fatal dose of pentobarbital sodium at 100 mg/kg. Prior to tissue extraction, the mice underwent cardiac perfusion through the left ventricle with 5 ml of PBS. The hearts were then meticulously harvested, dissected, and prepared for embedding.

**1.15 Hematoxylin and eosin (H&E) staining of aortic valves**

In summary, the sections of aortic valves underwent dewaxing and dehydration processes. Subsequently, the sections were subjected to staining with Harris' modified hematoxylin solution for a duration of 3 minutes, followed by washing with distilled water and counterstaining in eosin alcoholic solution for 1 minute. The sections were then dehydrated using escalating concentrations of ethyl alcohol and cleared in xylene for a duration of 2 minutes.

**1.16 Alizarin Red staining of aortic valves**

The aortic valve leaflets were subjected to fixation in 4% paraformaldehyde (PFA) overnight, followed by dehydration in 15% and 30% sucrose solutions, rinsing in distilled water, and staining with 2% Alizarin Red stain (Sigma-Aldrich) for 60 minutes. Subsequently, the valves underwent further rinsing in distilled water.

Quantitative analysis was conducted by measuring the area of Alizarin Red-positive fields using Image J software version 1.8 (National Institutes of Health). The results were averaged across independent biological replicates to obtain a mean value. Microscopic observations and image acquisitions were performed using an Olympus microscope, ensuring high-resolution visualization of the stained samples.

To determine the calcium concentration, a colorimetric assay based on the o-cresolphthalein complexone method was employed. This involved extracting calcium from cultured VICs using 0.1 mol/L hydrochloric acid. The calcium content in these extracts was then quantified by measuring the absorbance at the appropriate wavelength and converting it to micrograms of calcium per well in a 12-well plate, providing a direct measure of mineral deposition.

**1.17 Von Kossa staining of aortic valves**

The sections of aortic valve leaflets underwent a series of procedures including dewaxing, dehydration, and incubation with a 5% silver nitrate solution for 30 minutes. Subsequently, the sections were exposed to direct sunlight for 1 hour, followed by washing and treatment with a 5% sodium sulfate solution for 2 minutes. Following restaining with neutral red for an additional 3 minutes, the presence of calcium salt deposition in the aortic valve leaflets was analyzed using a light microscope. Mineralized nodules were isolated from the background by establishing a threshold value for reflection intensity using Image J 1.8 (National Institutes of Health), and the total area of mineralization was quantified.

**1.18 Masson’s trichrome staining of aortic valves**

The sections of aortic valves were first fixed in an acetone-methanol mixture for 10 minutes, then stained with Weigert iron hematoxylin for 5 minutes. After staining, the slides were rinsed under running tap water for 5 minutes to remove excess stain. Next, the sections were incubated in a solution of 0.02% azophloxine and 0.04% acid fuchsin for 20 minutes, followed by a rinse with a 1% acetic acid solution to prepare for the next staining step.

The slides were then treated with a 2.5% phosphotungstic acid solution for 10 minutes to enhance contrast, and again rinsed with the 1% acetic acid solution. Following this, the sections were dehydrated through a series of ethanol washes and cleared in xylene. The slides were finally mounted using a quick-hardening medium to secure the tissue sections in place.

To quantify collagen fibrosis, the blue-stained areas within the aortic valve region were measured using Image J software version 1.8 (National Institutes of Health), providing a quantitative assessment of the fibrotic changes in the valve tissue.

**1.19 Oil Red O staining of aortic valves**

Frozen sections of aortic valves were prepared and fixed with 4% paraformaldehyde for about 5 min. The fixed sections were gently washed 3 times with distilled water for 5 min each to remove fixative residue. Subsequently, the sections were immersed in Oil Red O staining solution and stained in the dark for 10 to 15 minutes. After staining, sections were slightly washed with 60% ethanol to remove excess staining solution and then washed three times with distilled water. For better visualization of nuclei, nuclei were counterstained with Mayer hematoxylin staining solution for 1 to 2 min, followed by slight differentiation with 1% hydrochloric acid solution and further rinsing with tap water for 10 min or blue stimulation with dilute lithium carbonate solution. Finally, the slices were dried in an oven at 60°C and sealed with glycerin gelatin or arabinan. Sealed sections were observed under a microscope and photographed to record Oil red O staining results. The lipid deposition were measured using Image J software version 1.8 (National Institutes of Health).

**1.20 RNA sequencing**

Total RNA was extracted from VICs using the Vazyme Total RNA Isolation Kit (RC112-01). The integrity of the RNA samples was evaluated, and cDNA libraries were prepared and sequenced with BGI's methodologies. Specifically, RNA sample quality was determined using an Agilent Bioanalyzer. For library construction, the TruSeq RNA Sample Preparation Kit (Illumina) was employed. Sequencing of each library was conducted using single-read technology on the HiSeq2000/1000 platform (Illumina). Quantitative gene expression analysis was performed in reads per kilobase per million mapped reads (RPKM) using the Cufflinks software. RNA-seq analyses were done by using BGI platform. For gene expression changes, Q value < 0.05 was used as the criteria. Log2FC, Q value for gene changes can be found in Excel File S1.

**1.21 Alkaline Phosphatase staining of VICs**

VICs were collected, rinsed three times in phosphate-buffered saline (PBS), and then treated with 4% paraformaldehyde for fixation. Following this, the cells were exposed to the BCIP/NBT substrate solution at ambient temperature in the absence of light for a duration of 15 minutes. Subsequent to removal of the substrate solution, images were captured utilizing a light microscope (Mshot), with alkaline phosphatase (ALP)-positive cells manifesting as a dark blue-violet hue. The Image-Pro software was utilized for the analysis of the positively stained region, employing the methodology described in a prior investigation.^36^

**1.22 Alizarin Red staining of VICs**

VICs were first washed with 1× phosphate-buffered saline (PBS) to remove any residual media. The cells were then fixed using a 4% paraformaldehyde (PFA) solution for 15 minutes at room temperature to preserve their structure. After fixation, the cells were gently rinsed with distilled water to remove excess PFA. Subsequently, the fixed cells were stained with a 0.2% Alizarin Red solution (Servicebio, G1038-100ML) for 30 minutes to visualize mineralization. Finally, the stained cells were washed with distilled water to remove any unbound stain, preparing them for further analysis or imaging. Analysis was performed as previously described.

**1.23 Efflux of cholesterol**

VICs were exposed to cholesterol for a duration of 8 hours following various treatments, subsequently undergoing PBS washes and medium replacement. The cholesterol levels in both the culture medium and cell lysates were quantified by using cholesterol assay kit (F002-1-1, Jiancheng Bio., Nanjing, China) after 4 hours. The cholesterol efflux rate was determined by calculating the ratio of cholesterol present in the medium to that in the cell lysate as described in a previous study.^37^

**1.24 Chromatin immunoprecipitation sequencing (CHIP-seq)**

The CHIP assay was conducted by Bioyigene Biotechnology in Wuhan, China. In summary, VICs were transfected with Ad-FOXS1 for a period of 72 hours. Post-transfection, the VICs were collected and subjected to cross-linking using 1% paraformaldehyde for 10 minutes at room temperature. The cross-linking was quenched by adding 125 mM glycine and incubating for an additional 5 minutes. The cells were lysed, and the nuclei were pelleted by centrifugation at 2000g for 5 minutes. The nuclear pellets were then lysed further and sonicated to shear the chromatin DNA into fragments. A portion (10%) of the sheared chromatin was reserved as the "input" control, while the remainder (90%) was used for immunoprecipitation with a Flag-tag antibody, denoted as "IP". DNA from both the input and IP samples was purified using a phenol-chloroform extraction method. High-throughput DNA sequencing libraries were prepared with the VAHTS Universal DNA Library Prep Kit for Illumina V3 (Catalog NO. ND607, Vazyme). The library fragments within the size range of 200-500 base pairs were enriched, quantified, and sequenced on a Novaseq 6000 sequencer (Illumina) using the PE150 model. The sequencing and data analysis services were provided by Bioyi Biotechnology Co., Ltd. in Wuhan. The CHIP-seq data have been deposited and are accessible in the Sequence Read Archive (SRA) database under accession number PRJNA1126021.

**1.25 Luciferase reporter assay**

DNA transfections and subsequent luciferase assays were performed in accordance with the protocol of the Dual Luciferase Reporter Assay System. HEK 293T cells were seeded in 12-well plates at a density of 1×10^5 cells per well and cultured for one day before transfection. The Renilla plasmid (p-RL-TK) was co-transfected with the relevant vectors, and the firefly luciferase activity was measured concurrently from each sample. The PPRE-Luc vector was transfected in the presence of either Ad-NC or Ad-FOXS1 adenoviruses. After transfection, the cells were collected, and luciferase activities were quantified.

To identify the FOXS1 binding site on the BSCL2 promoters, a genomic fragment spanning nucleotides 62709825 to 62709845 (AGTGATTCTTCGTTATGGCCG) from the BSCL2 promoter region on chromosome 11 was amplified by PCR and cloned into the pGL4.16 vector. Additionally, a sequence containing the nucleotides TTTTTTTCCCCCCTTTTTTT was inserted into pGL4.16 to serve as a negative control. The recombinant pGL vectors were then transfected into HEK293T cells, either with or without FOXS1 overexpression. Luciferase activity was measured and analyzed as previously described to assess the transcriptional activity and FOXS1 binding to the BSCL2 promoter region.

**1.26 Antibodies and reagents**

The following antibodies were used for western blotting, immunohistochemistry and immunofluorescence: FOXS1 (Proteintech, 16234-1-AP), RUNX2 (CST, 8486), ALP (R&D, MAB1448), GAPDH (Proteintech, 60004-1-Ig), Flag-tag (Proteintech, 66008-1-Ig), ABCA1 (Abcam, ab18180), ABCG1 (Proteintech, 13578-1-AP), Vimentin (Proteintech, 60330-1-Ig), NLRP3 (Abcam, ab263899), IL-1β (Abcam, ab9722), Caspase-1 (Abcam, ab207802), PPARγ (Proteintech, 66936-1-Ig), LXRα (Proteintech, 14351-1-AP), BSCL2 (Abclonal, A14583), goat anti-rabbit IgG (ab150077, 1:200 dilution), goat anti-mouse IgG (ab150115, 1:200 dilution). Human oxLDL (YB-002, Yiyuan Biotech.), Bodipy-Choleterol (810255P, Avanti Polar Lipids), Cholesterol (ST1155, Beyotime Biotech.), MCC950 (GC31644, GlpBio Inc.), IMM-H007 (S4460, Selleck). Mouse recombinant Bscl2 (RG505984, Abmart).

**1.27 Statistical analysis**

Statistical analysis was performed using GraphPad Prism 8 (GraphPad Software, Inc.) and presented as mean ± standard deviation (SD). Except where otherwise noted, all quantitative studies were performed at least in six replications. The graph dots represent independent individual biological replicates. The normality of the data was confirmed using the Shapiro-Wilk test. Parametric tests, such as unpaired Student’s t-tests or one-way ANOVA, were employed if the data passed the normality test (alpha = 0.05). Non-parametric tests, specifically the Mann-Whitney test, were utilized if the data did not pass the normality test. The link between 2 variables was assessed using two-tailed Pearson correlation analysis. Statistical significance was defined as P < 0.05.

**1.28 Data availability statement**

The bulk RNA-seq data and scRNA-seq data were obtained from the Gene Expression Omnibus (GEO) database (GSE148219, GSE76718, GSE153555, GSE180278). The CHIP-seq data have been uploaded and available on the Sequence Read Archive (SRA) database (PRJNA1126021). The data, methods used in the analysis, and materials will be made available to any researcher for purposes of reproducing the results or replicating the procedure on reasonable request to the corresponding author.

1. **Supplemental** **figures and legends**

**Figure S1**


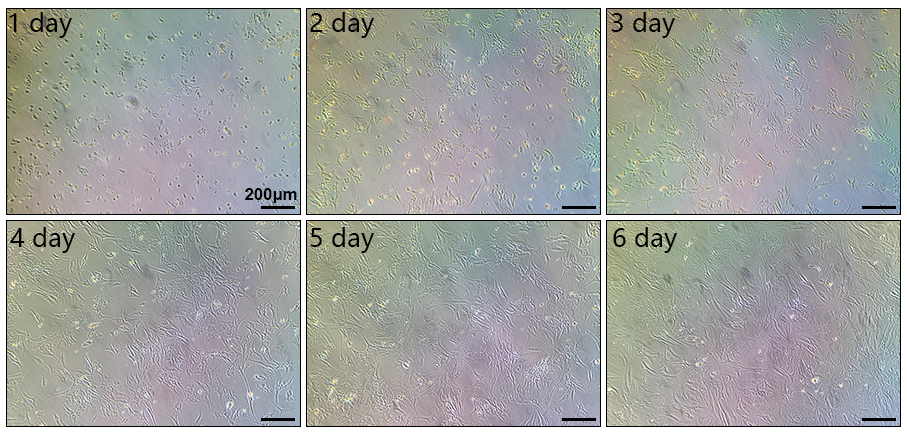


**Figure S1. Growth process and morphology of cultured primary VICs.**

Optical microscopy was used to observe the growth process and morphological changes of primary VICs from day 1 to day 6 after implantation. VICs initially present as small dot - like distributions, and then gradually proliferate and form spindle shapes, similar to fibroblast - like cells. Scale bar = 200μm.

**Figure S2**

**
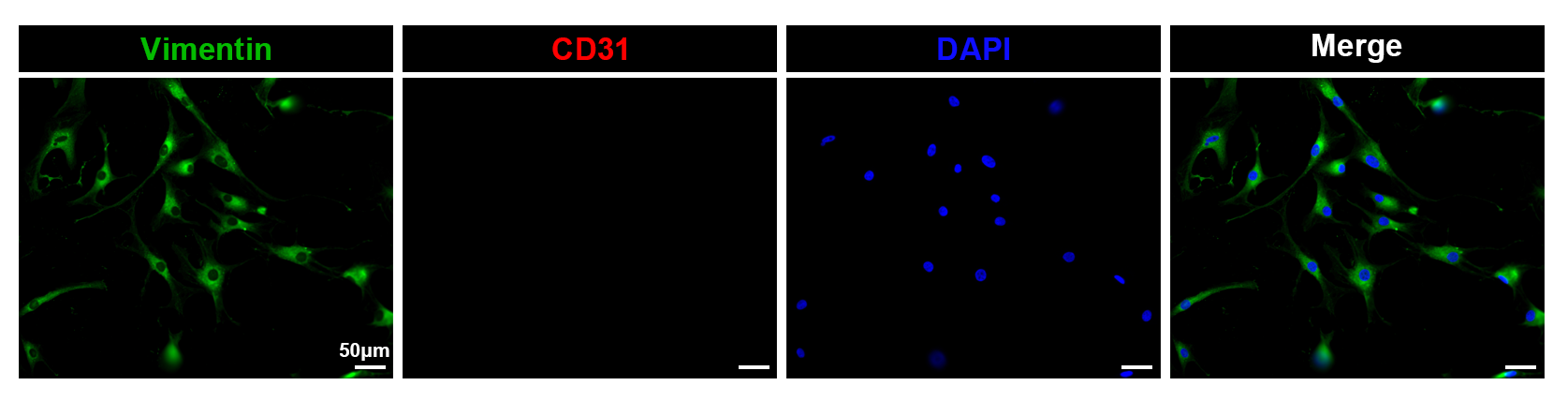
**

**Figure S2. Identification of biomarkers in cultured primary VICs.**

Immunofluorescence staining of Vimentin (green) and CD31 (red) in VICs. DAPI was used for nuclear counterstaining (blue). Scale bar = 50μm.

**Figure S3**


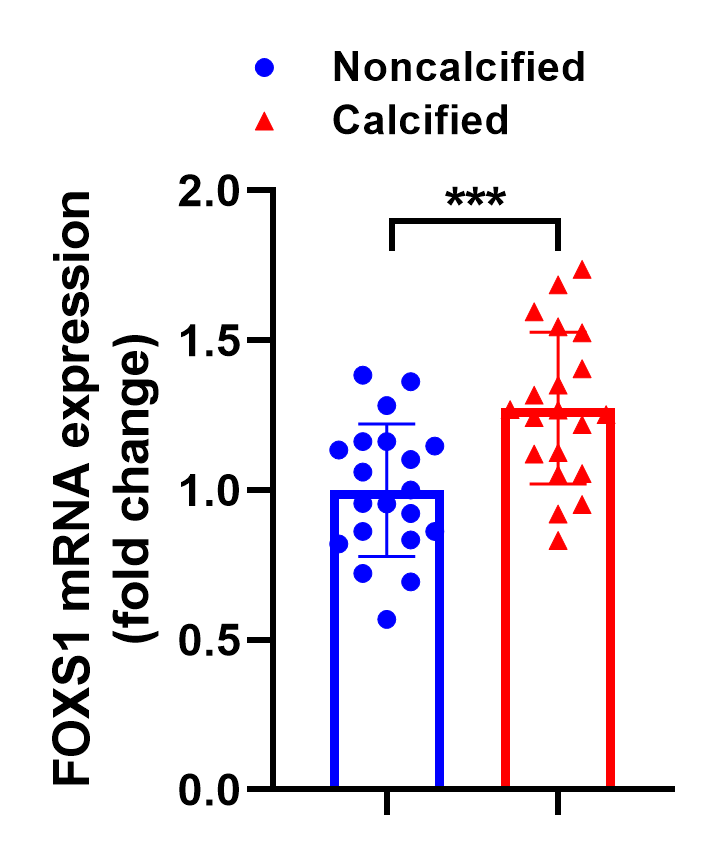


**Figure S3. The mRNA of FOXS1 upregulated in calcified aortic valves.**

qRT-PCR analysis of FOXS1 in noncalcified and calcified aortic valves. Unpaired two-tailed Student’s t-test (n=20). Values are the mean ± SD. *P<0.05, **P<0.01, ***P<0.001.

**Figure S4**


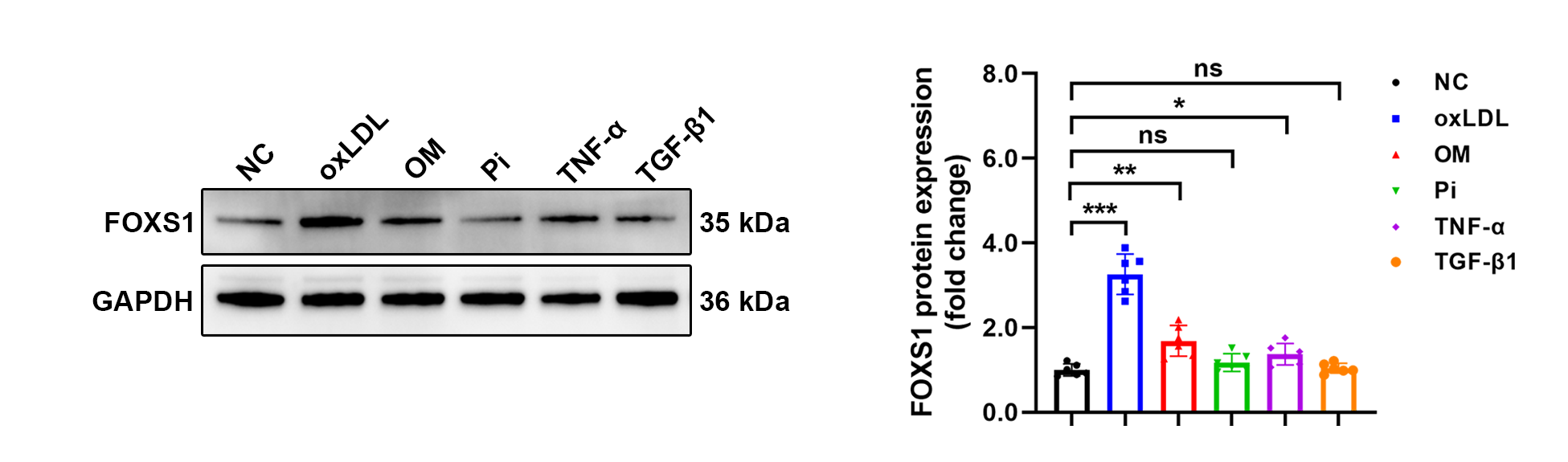


**Figure S4. Comparison of the activation of FOXS1 by various osteogenic inducing stimulation.**

Western blot analysis the expression level of FOXS1 in VICs treated with different stimulation (n=6). Values are the mean ± SD. Data were analyzed using one-way ANOVA followed by Bonferroni multiple comparisons test. *P<0.05, **P<0.01, ***P<0.001.

**Figure S5**


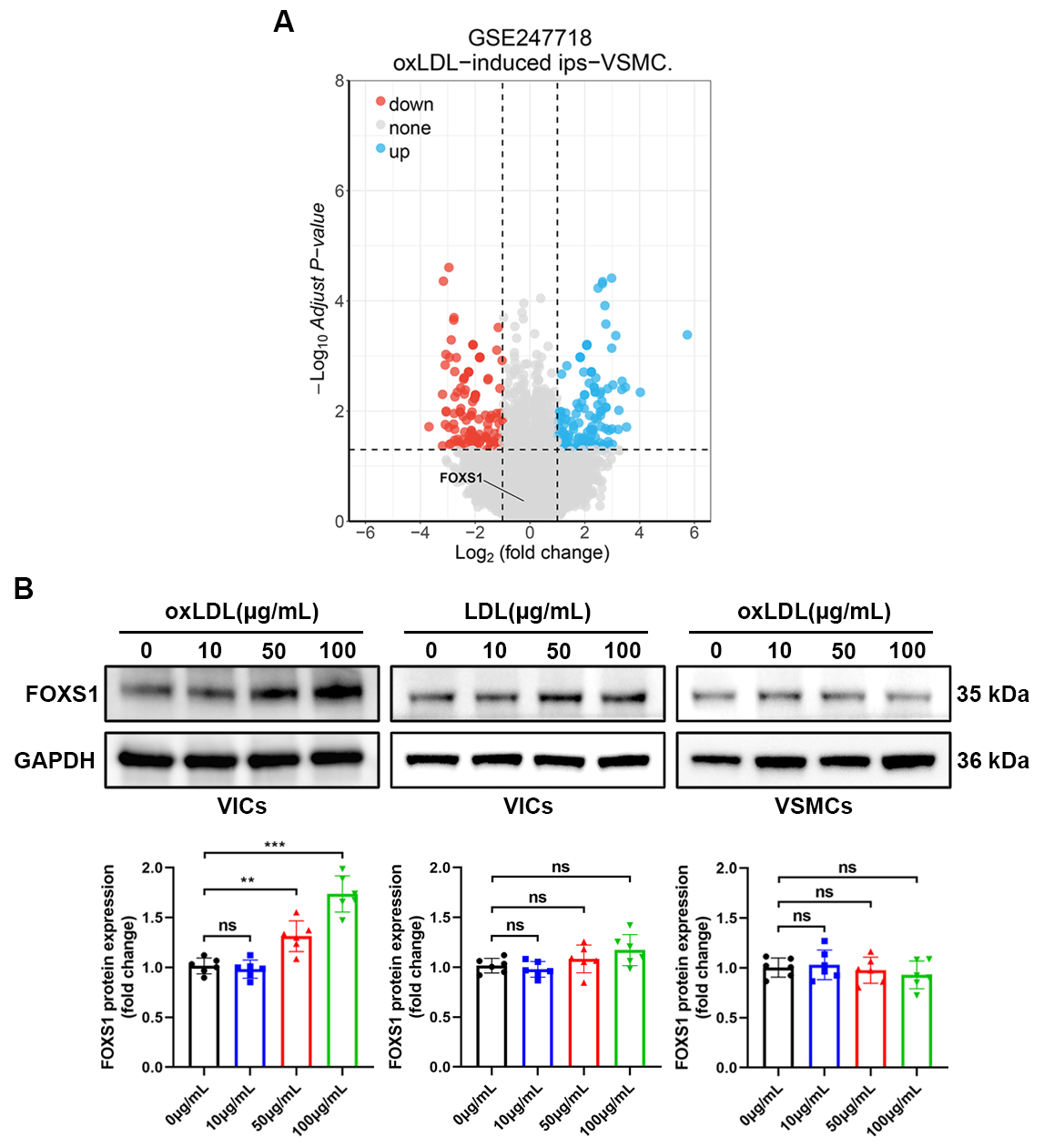


**Figure S5. The expression level of FOXS1 in VICs infected with different MOI of adenovirus.**

Values are the mean ± SD. Data were analyzed using one-way ANOVA followed by Bonferroni multiple comparisons test, n=6. *P<0.05, **P<0.01, ***P<0.001.

**Figure S6**


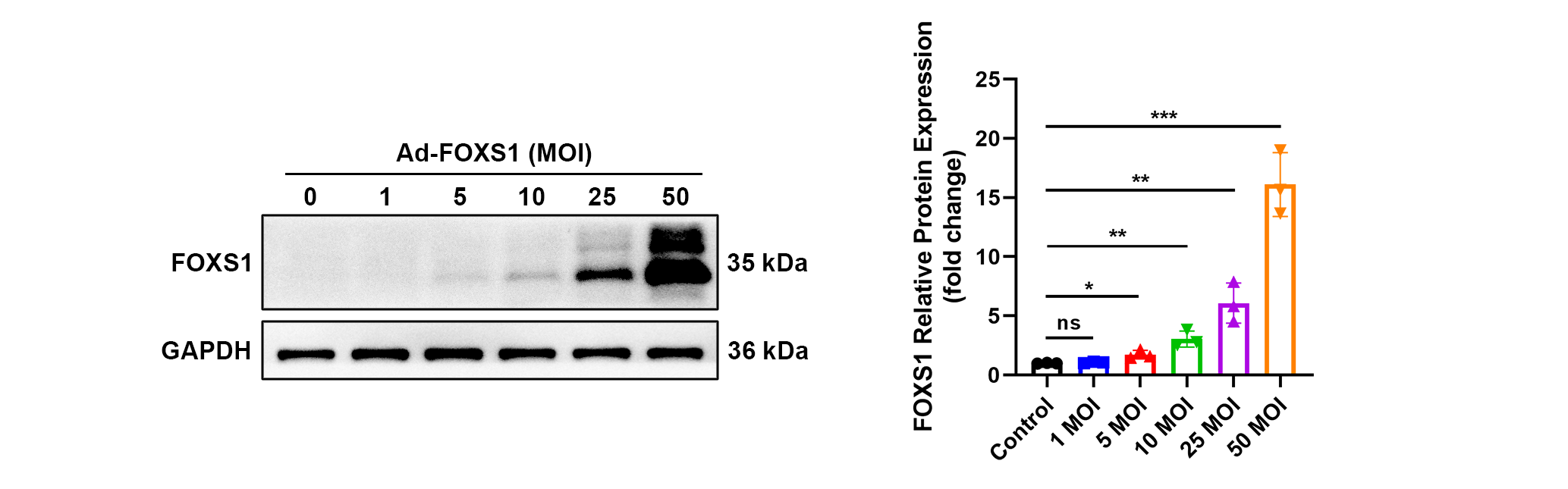


**Figure S6. The expression level of FOXS1 in VICs infected with different MOI of adenovirus.**

Values are the mean ± SD. Data were analyzed using one-way ANOVA followed by Bonferroni multiple comparisons test, n=6. *P<0.05, **P<0.01, ***P<0.001.

**Figure S7**


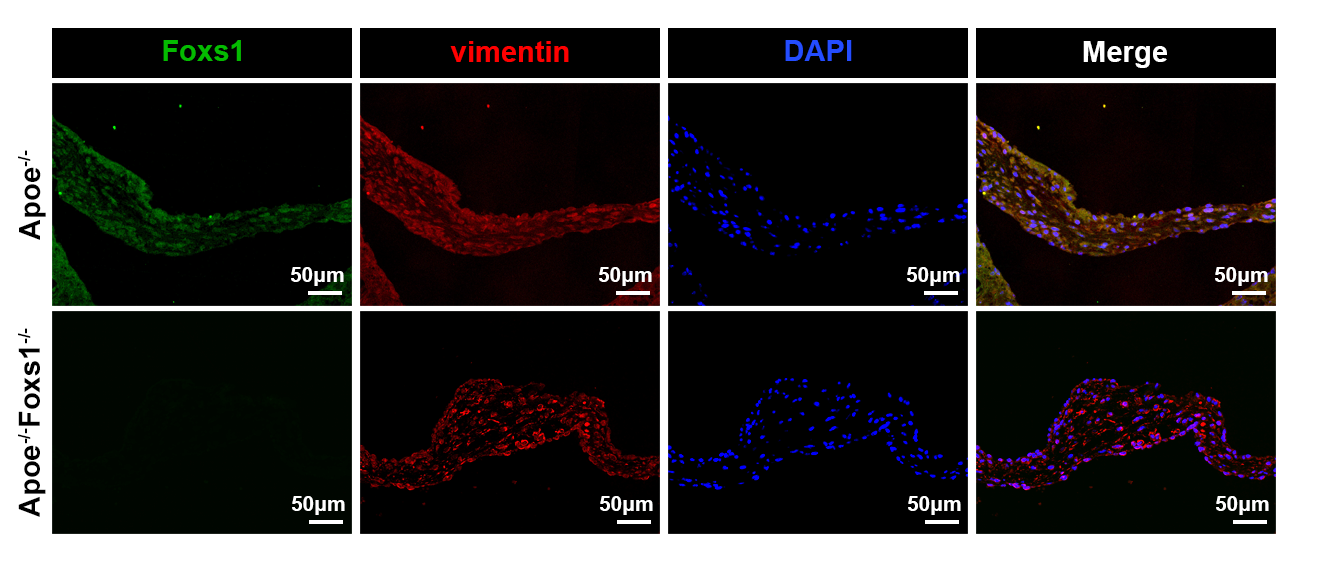


**Figure S7. Validation of Foxs1 knockout efficiency in Apoe^-/-^ mice.**

Immunofluorescence staining of Foxs1 (green) and vimentin (red) in the aortic valves of Apoe^-/-^ and Apoe^-/-^Foxs1^-/-^ mice. Scale bar=50μm. DAPI was used for nuclear counterstaining (blue).

**Figure S8**


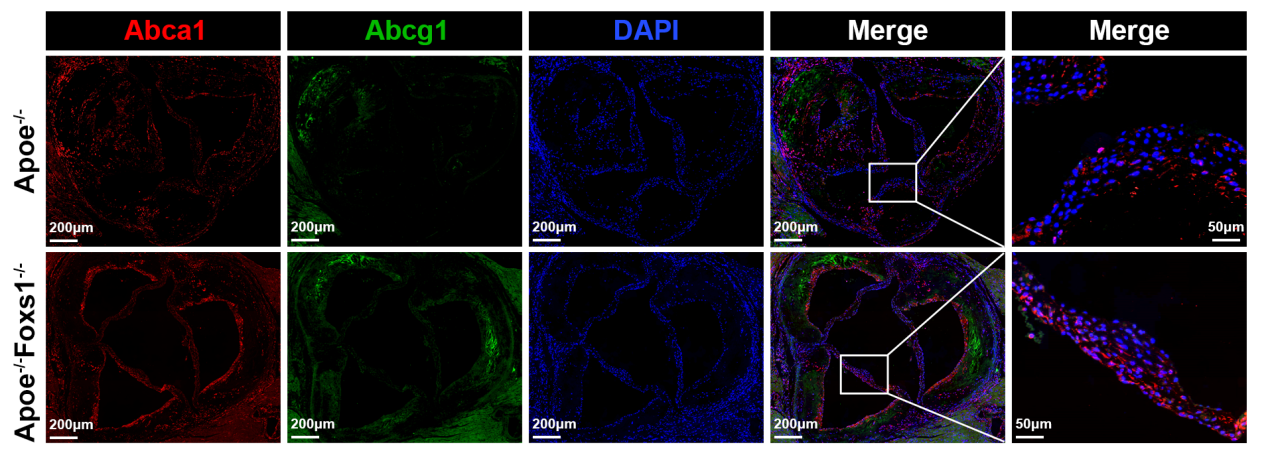


**Figure S8. Abca1 and Abcg1 were upregulated in the aortic valves of Foxs1 knockout mice.**

Immunofluorescence staining of Abca1 (red) and Abcg1 (green) in the aortic valves of Apoe^-/-^ and Apoe^-/-^Foxs1^-/-^ mice. DAPI was used for nuclear counterstaining (blue).

**Figure S9**


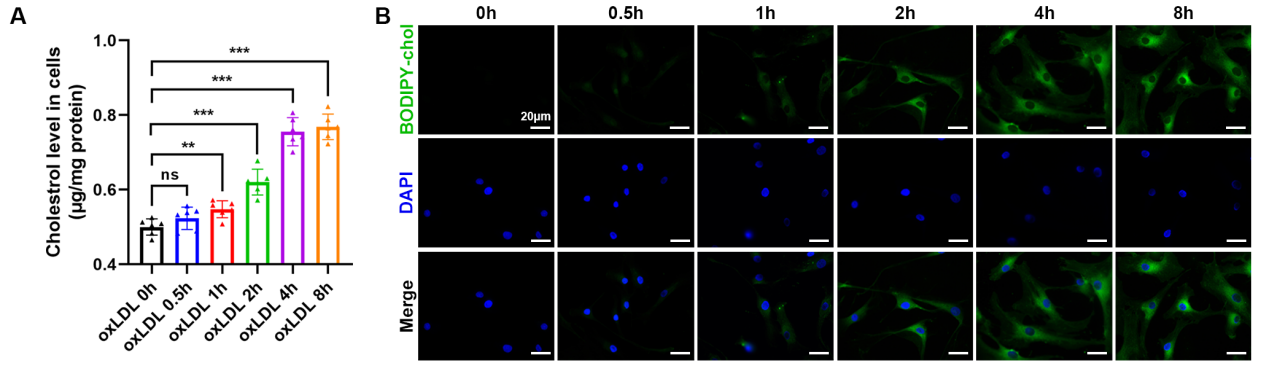


**Figure S9. oxLDL increased cholesterol levels in VICs.**

(A) Determination of cholesterol level in VICs treated with oxLDL at different time point (n=6). (B) VICs were incubated with Bodipy-cholesterol (1μM). Representative images of Bodipy-cholesterol-labeled VICs at different time point are shown. Scale bar = 20 μm. DAPI was used for nuclear counterstaining (blue). Data were analyzed by one-way ANOVA followed by Bonferroni multiple comparisons test (A). Values are the mean ± SD. *P<0.05, **P<0.01, ***P<0.001.

**Figure S10**


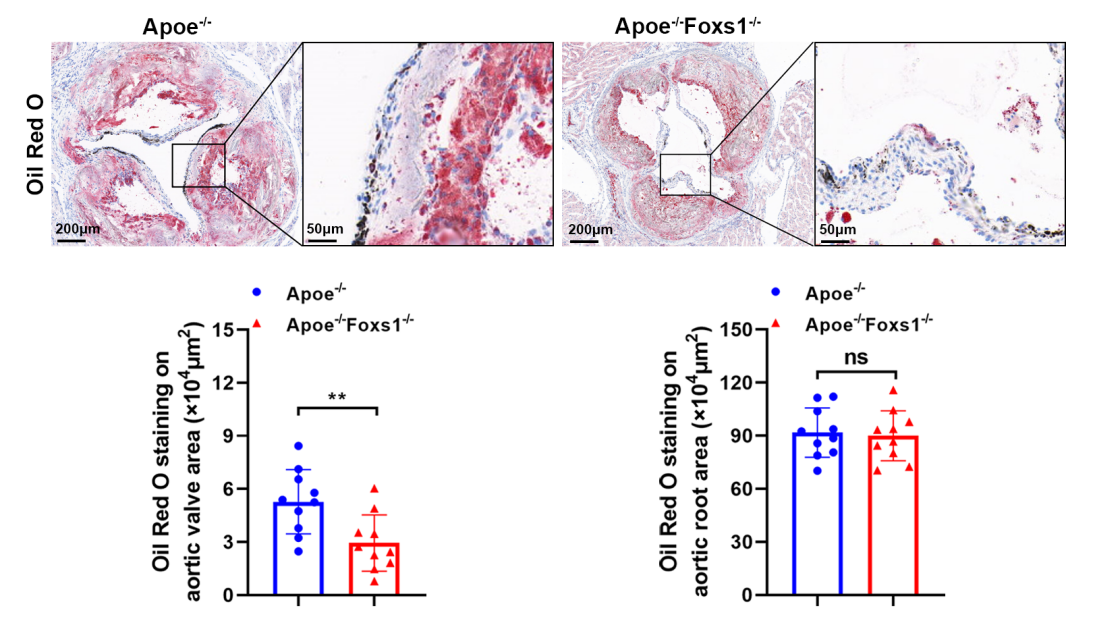


**Figure S10. FOXS1 deficiency attenuated lipid deposition in aortic valves of Apoe^-/-^ mice.**

Oil Red O staining of aortic valves and root area from Apoe^-/-^ and Apoe^-/-^Foxs1^-/-^ mice fed with HFD (n=10). Data were analyzed by unpaired two-tailed Student’s t-test. Values are the mean ± SD. *P<0.05, **P<0.01, ***P<0.001.

**Figure S11**

**
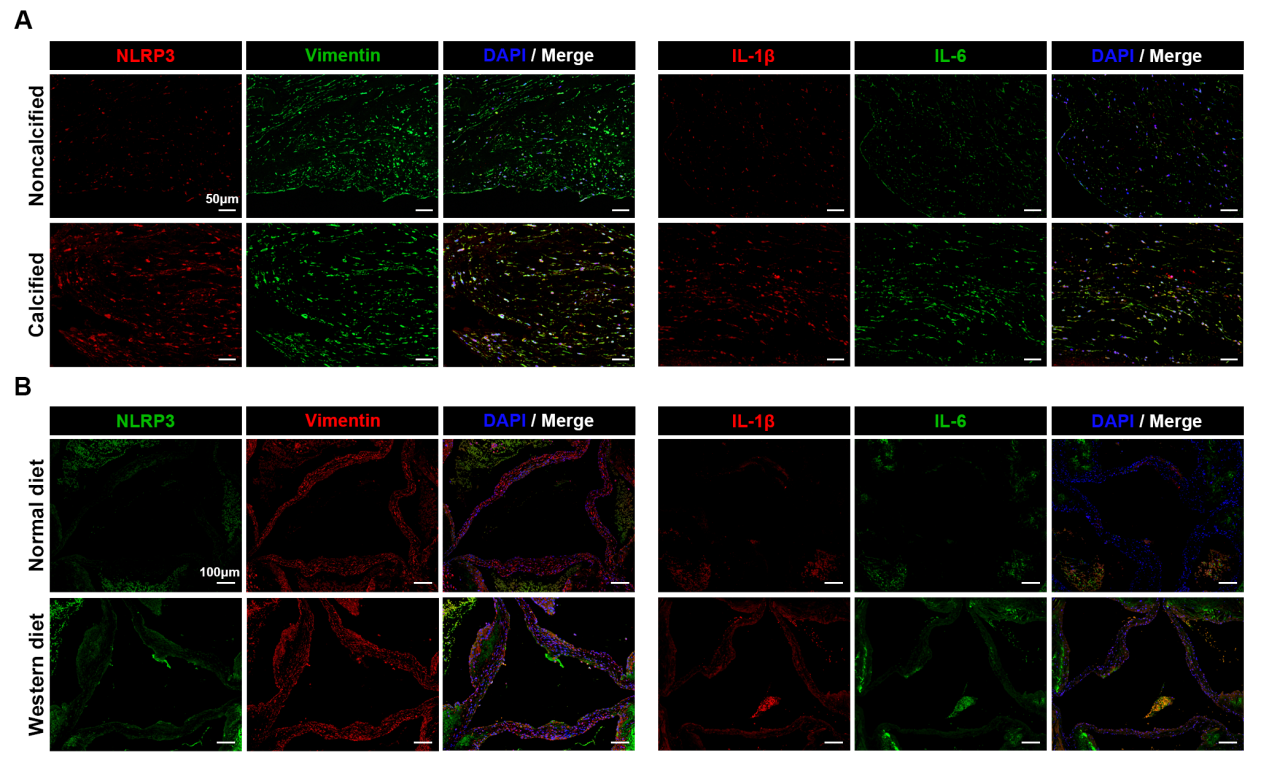
**

**Figure S11. NLRP3, IL-1β and IL-6 were upregulated in the calcified aortic valves of human and mice.**

(A) Immunofluorescence staining of NLRP3 (red), Vimentin (green) or IL-1β (red) and IL-6 (green) in human aortic valves. Scale bar = 50μm. (B) Immunofluorescence staining of NLRP3 (green), Vimentin (red) or IL-1β (red) and IL-6 (green) in the aortic valves of Apoe^-/-^ mice. Scale bar = 100μm. DAPI was used for nuclear counterstaining (blue).

**Figure S12**


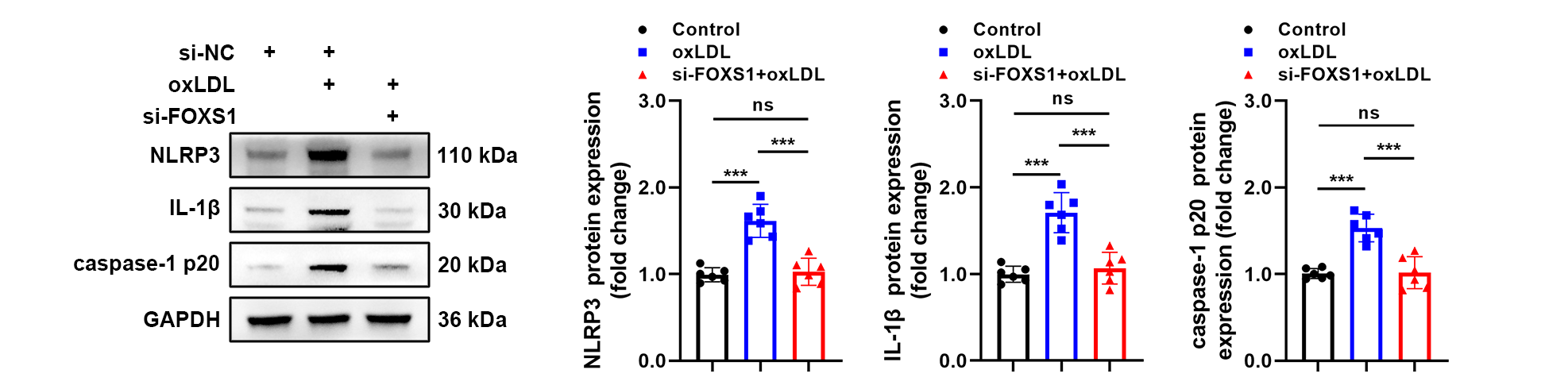


**Figure S12. FOXS1 mediates oxLDL-induced NLRP3 inflammasome activation.**

Western blot analysis of protein levels of VICs treated with oxLDL and si-FOXS1 (n=6). Data were analyzed using one-way ANOVA followed by Bonferroni multiple comparisons test. Values are the mean ± SD. *P<0.05, **P<0.01, ***P<0.001.

**Figure S13**


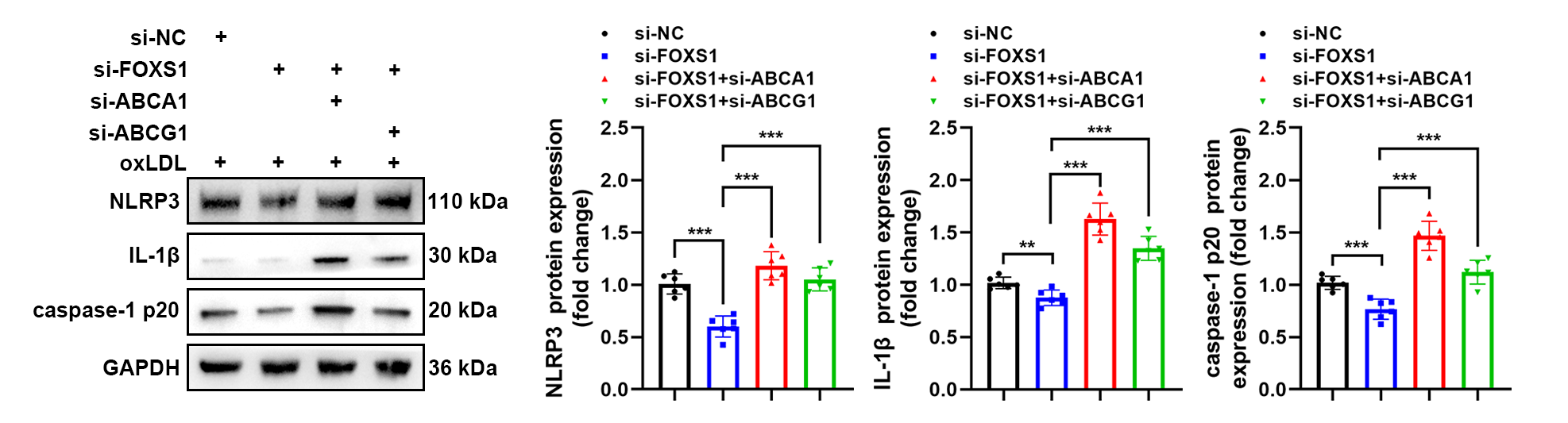


**Figure S13. FOXS1 mediates NLRP3 inflammasome activation via ABCA1 and ABCG1.**

Western blot analysis of protein levels of VICs treated with si-FOXS1 and si-ABCA1 or siABCG1 (n=6). Data were analyzed using one-way ANOVA followed by Bonferroni multiple comparisons test. Values are the mean ± SD. *P<0.05, **P<0.01, ***P<0.001.

**Figure S14**


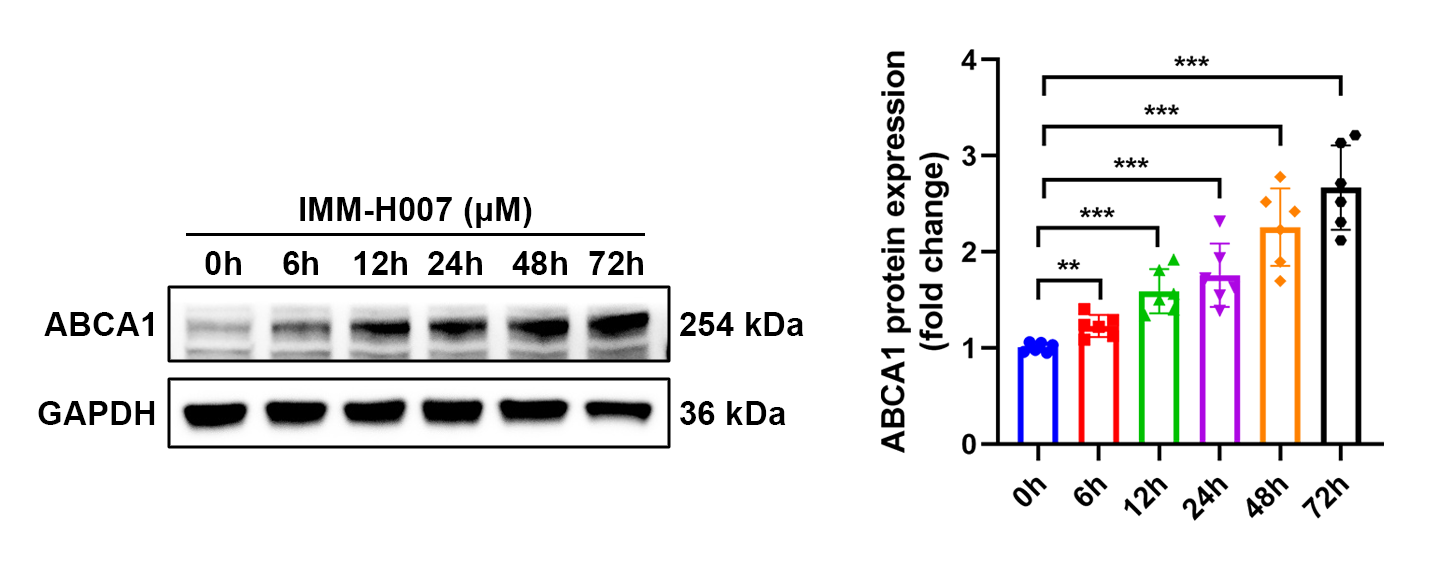


**Figure S14. The expression of ABCA1 increased over the duration of IMM-H007 treatment.**

The Western blot analysis of ABCA1 in VICs treated with IMM-H007 in different time point. One-way ANOVA followed by Bonferroni multiple comparisons test (n=6). Values are the mean ± SD. *P<0.05, **P<0.01, ***P<0.001.

**Figure S15**


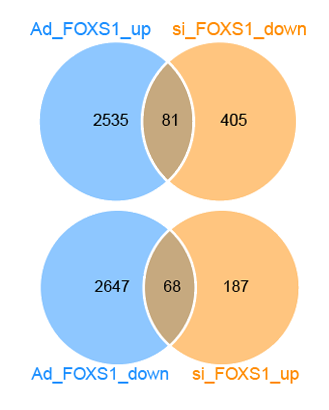


**Figure S15. Identification of the DEGs that positively or negatively regulated by FOXS1 in RNA-seq.**

The up-regulated genes in the Ad-FOXS1 group overlapped with the down-regulated genes in the si-FOXS1 group, resulting in 81 DEGs that were positively regulated by FOXS1. The down-regulated genes in the Ad-FOXS1 group overlapped with the up-regulated genes in the si-FOXS1 group, resulting in 68 DEGs that were positively regulated by FOXS1. A total of 149 DEGs were obtained that positively or negatively regulated by FOXS1.

**Figure S16**


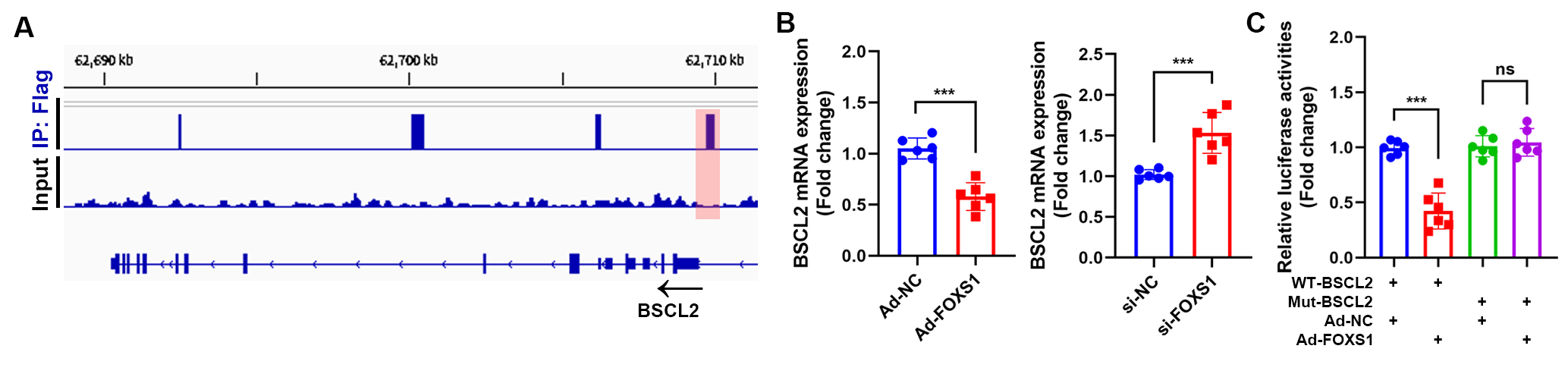


**Figure S16. FOXS1 regulates BSCL2 expression by inhibiting its promoter.**

(A) IGV tracks showing FOXS1 CHIP-seq signals at BSCL2 gene locus between Input and IP-Flag in VICs. (B) qRT-PCR analysis of BSCL2 in VICs treated with Ad-FOXS1 and si-FOXS1 (n=6). (C) Luciferase activation driven by the wild type or mutant BSCL2 promoter after normalization to Renila luciferase in HEK-293 cells (n=6). Values are the mean ± SD. P values were calculated using unpaired two-tailed Student’s t-test (B) or one-way ANOVA followed by Bonferroni multiple comparisons test (C). *P<0.05, **P<0.01, ***P<0.001.

**Figure S17**


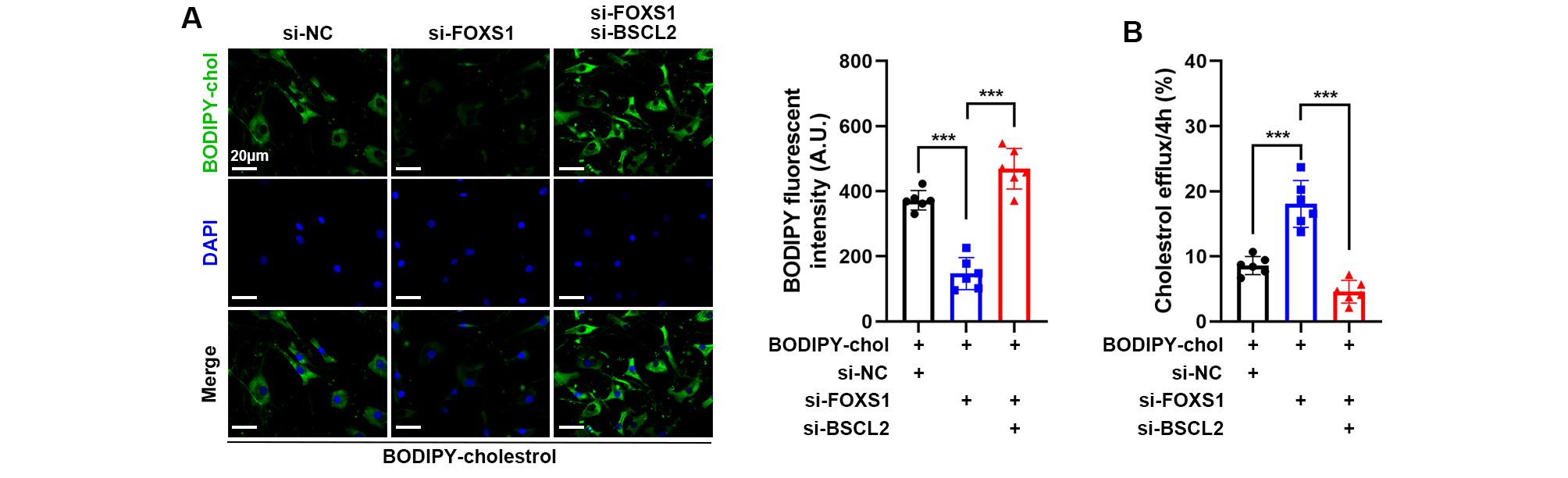


**Figure S17. FOXS1 mediates VICs cholesterol efflux through BSCL2.**

(A) Representative images of Bodipy-cholesterol-labeled VICs treated with si-FOXS1 and si-BSCL2. (B) Cholesterol efflux of VICs. Data were analyzed using one-way ANOVA followed by Bonferroni multiple comparisons test (n=6). Scale bar=20μm. Values are the mean ± SD. *P<0.05, **P<0.01, ***P<0.001.

**Figure S18**


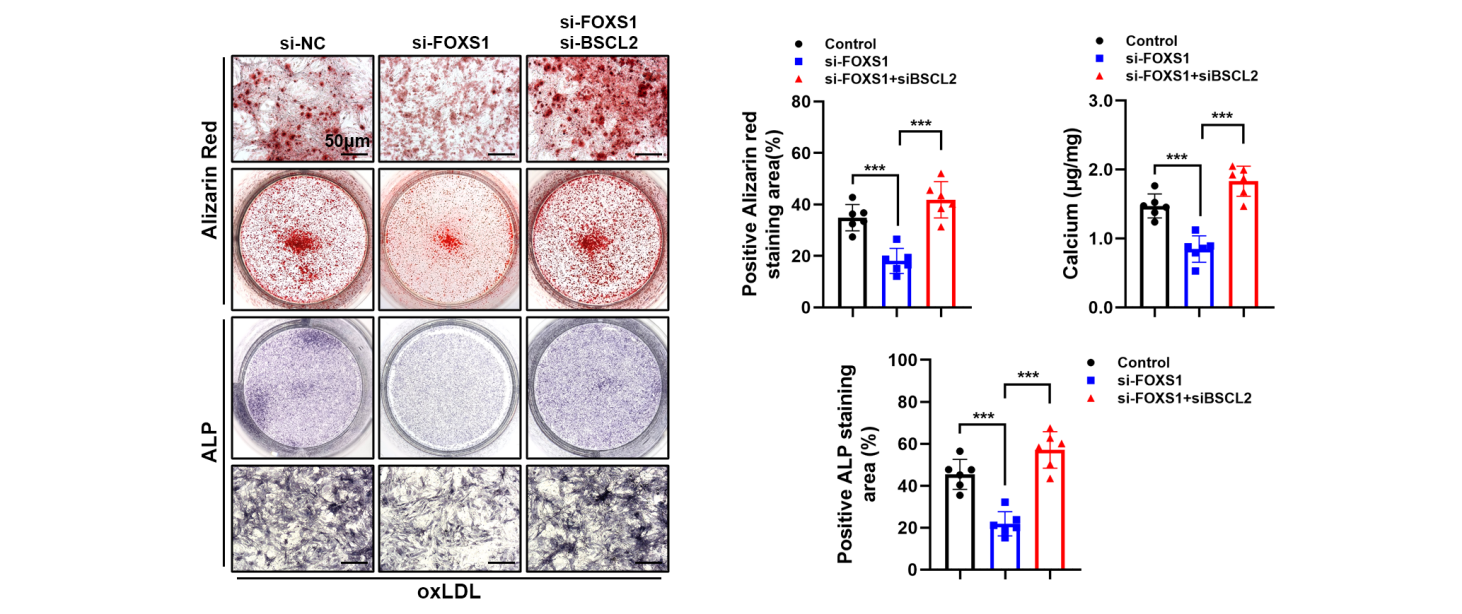


**Figure S18. FOXS1 promotes calcium salt deposition and ALP level through BSCL2.**

Alizarin red staining, calcium content and ALP staining of VICs treated with si-FOXS1 and si-BSCL2 (n=6). Scale bar=50μm. Values are the mean ± SD. Data were analyzed using one-way ANOVA followed by Bonferroni multiple comparisons test. *P<0.05, **P<0.01, ***P<0.001.

**Figure S19**


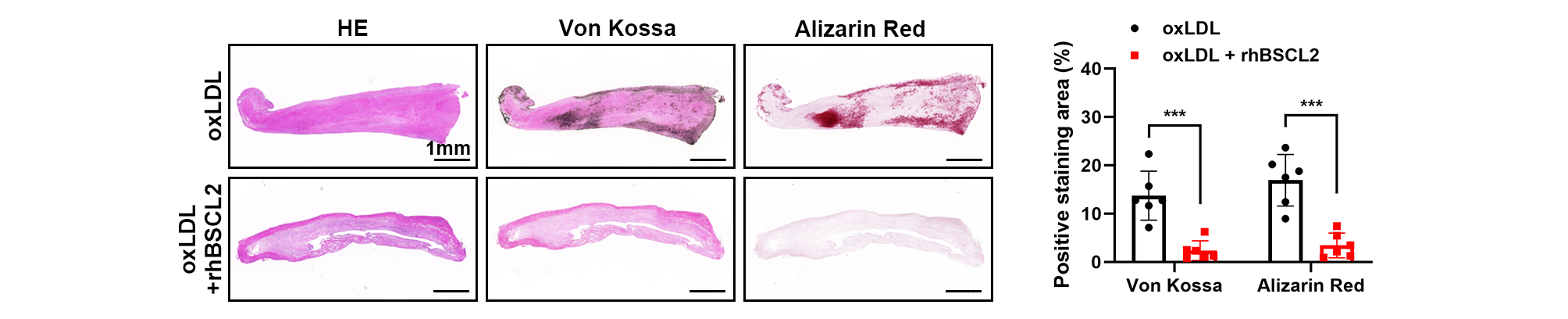


**Figure S19. BSCL2 inhibits aortic valve calcification in vitro.**

HE, Von kossa and Alizarin Red staining of aortic valves treated with rhBSCL2 and oxLDL for 21 days in vitro (n=6). Scale bar=1mm. Values are the mean ± SD. Data were analyzed using one-way ANOVA followed by Bonferroni multiple comparisons test. *P<0.05, **P<0.01, ***P<0.001.

**Figure S20**


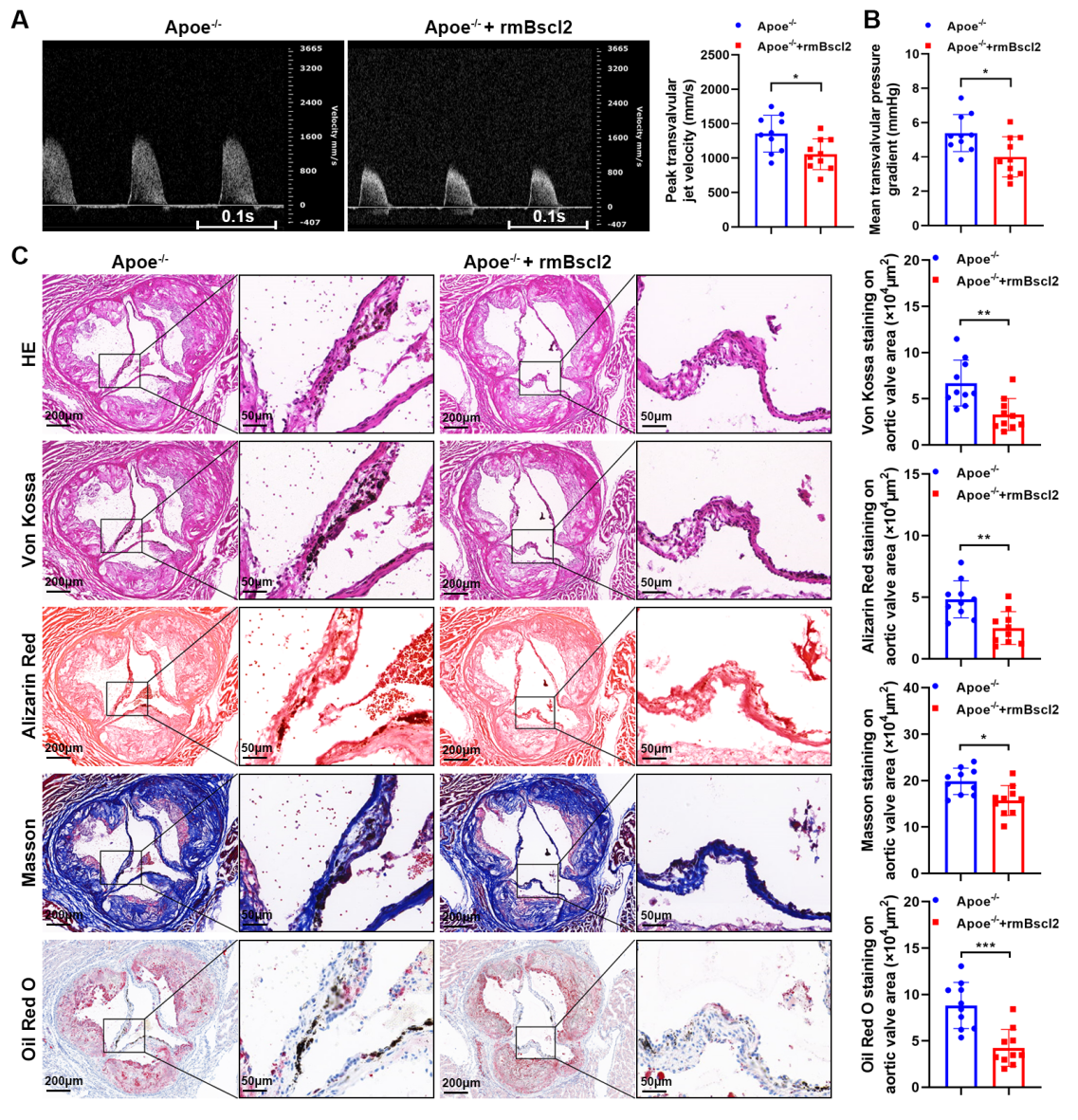


**Figure S20. BSCL2 mitigates aortic valve calcification in vivo.** (A and B) The severity of aortic valve stenosis in Apoe^-/-^ and Apoe^-/-^ + rmBscl2 mice fed with HFD was evaluated using echocardiography. Pulsed-wave Doppler examination was conducted across the aortic valve to measure peak transvalvular jet velocity (A) and the mean transvalvular pressure gradient (B). (C) HE, Von kossa, Alizarin Red and Masson and Oil Red O staining of aortic valves from Apoe^-/-^ and Apoe^-/-^ + rmBscl2 mice fed with HFD. Values are the mean ± SD. Unless otherwise indicated, data were analyzed by unpaired two-tailed Student’s t-test (n=10). *P<0.05, **P<0.01, ***P<0.001.

**Figure S21**


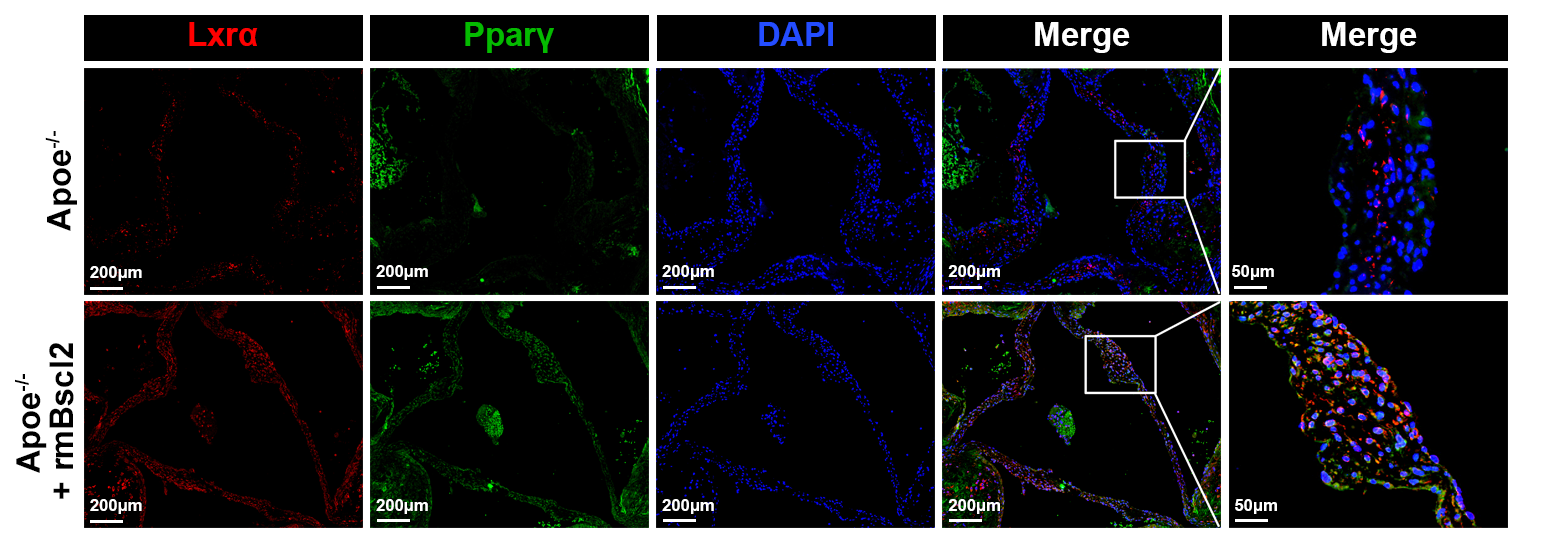


**Figure S21.** **Lxrα and Pparγ were upregulated in the aortic valves of mice with rmBscl2.**

Immunofluorescence staining of Lxrα (red) and Pparγ (green) in the aortic valves of Apoe^-/-^ and Apoe^-/-^ + rmBscl2 mice.

1. **Supplementary Tables**

**Table S1. Clinical characteristics of patients for qRT-PCR and Western blot analysis**

| **Parameters** | **Normal** | **CAVD** | P value |
| --- | --- | --- | --- |
|  | **(n=20)** | **(n=20)** |  |
| **Age, y** | 55 ± 6 | 58 ± 7 | 0.919 |
| **Male, n (%)** | 9(45.0) | 12(60.0) | 0.342 |
| BMI (kg/m2) | 21.43 ± 0.62 | 22.04 ± 0.76 | 0.265 |
| **Diagnose** | DCM | CAVD |  |
| **Hypertension, n (%)** | 11(55.0) | 13(65.0) | 0.519 |
| **Triglycerides (mmol/L)** | 1.48 ± 0.12 | 1.54 ± 0.16 | 0.172 |
| **LDL (mmol/L)** | 1.89 ± 0.18 | 1.98 ± 0.21 | 0.402 |
| **HDL (mmol/L)** | 1.76 ± 0.23 | 1.68 ± 0.26 | 0.852 |
| **Diabetes mellitus, n (%)** | 5(25.0) | 8(40.0) | 0.311 |
| **Smoking, n (%)** | 12(60.0) | 14(70.0) | 0.507 |
| **Statins, n (%)** | 11(55.0) | 13(65.0) | 0.519 |
| **β-Blockers, n (%)** | 6(30.0) | 9(45.0) | 0.327 |
| **ACEi/ARB, n (%)** | 7(35.0) | 10(50.0) | 0.337 |
| **LVEF (%)** | 55 ± 6.7 | 54 ± 8.2 | 0.675 |
| **Transvalvular pressure gradient (mmHg)** | 15.36 ± 2.68 | 84.51 ± 18.48 | <0.0001 |
| AVA (cm2) | 3.18 ± 0.42 | 0.81 ± 0.19 | <0.0001 |

ACEi, angiotensin-converting enzyme inhibitor; ARB, angiotensin receptor blocker; AVA, aortic valve area; BMI, Body mass index; CAVD, calcific aortic valve disease; CAVs, calcific aortic valves; DCM, dilated cardiomyopathy; HDL, high-density lipoprotein cholesterol; LDL, low-density lipoprotein cholesterol; LVEF, left ventricular ejection fraction. Values are mean ± standard deviation (SD) or %. Statistical differences were determined using two-tailed unpaired Student’s t-test.

**Table S2. Primes for qRT-PCR and target sequences of siRNAs.**

| **Primers for qRT-PCR** | **Primer sequence (5′ to 3′)** |
| --- | --- |
| **GAPDH forward** | GGAGTCCACTGGCGTCTTCA |
| **GAPDH reverse** | GTCATGAGTCCTTCCACGATACC |
| **BCL11B forward** | GGTGCCTGCTATGACAAGG |
| **BCL11B reverse** | GGCTCGGACACTTTCCTGAG |
| **EBF2 forward** | TAGGAAGAGGACCAACTCTGAAA |
| **EBF2 reverse** | CGACATTAGCGTCCACCACTC |
| **FHL2 forward** | GTACAGACTGCTATTCCAACGAG |
| **FHL2 reverse** | GCACTGCATGGCATGTTGTT |
| **FOXS1 forward** | GCTACATCATGGGCCGATTC |
| **FOXS1 reverse** | CAAACATGTCGTGGCAGTCA |
| **GLI3 forward** | GAAGTGCTCCACTCGAACAGA |
| **GLI3 reverse** | GAAGTGCTCCACTCGAACAGA |
| **HEY1 forward** | GTTCGGCTCTAGGTTCCATGT |
| **HEY1 reverse** | CGTCGGCGCTTCTCAATTATTC |
| **NR2F2 forward** | TCATGGGTATCGAGAACATTTGC |
| **NR2F2 reverse** | TTCAACACAAACAGCTCGCTC |
| **PHLDA2 forward** | GCGAGGGCGAGTTGGAGAA |
| **PHLDA2 reverse** | CCACCTTGAGGATGGAGTGGAA |
| **POU2AF1 forward** | TGGACACCTTACACCGAGTAT |
| **POU2AF1 reverse** | CGGAGAGGCATAGGTCAACAC |
| **RFX8 forward** | TGGACACCTTACACCGAGTAT |
| **RFX8 reverse** | CGGAGAGGCATAGGTCAACAC |
| **RUNX1 forward** | CTGCCCATCGCTTTCAAGGT |
| **RUNX1 reverse** | GCCGAGTAGTTTTCATCATTGCC |
| **RUNX2 forward** | CTGTCATGGCGGGTAACGAT |
| **RUNX2 reverse** | GGGTTCCCGAGGTCCATCTA |
| **SOX17 forward** | GTGGACCGCACGGAATTTG |
| **SOX17 reverse** | GGAGATTCACACCGGAGTCA |
| **VDR forward** | GTGGACATCGGCATGATGAAG |
| **VDR reverse** | GGTCGTAGGTCTTATGGTGGG |
| **ABCA1 forward** | AGGCTTGTCAAGGGGTAGGA |
| **ABCA1 reverse** | AGCAGCAGCTGACATGTTTGT |
| **ABCA2 forward** | CCTCATCAAGACAGGGCGTT |
| **ABCA2 reverse** | TCACACTCTGGCTGCTCTTG |
| **ABCA6 forward** | ACTGCCCTCAAGAGAACGTG |
| **ABCA6 reverse** | CCAGGAGCAAGACAGGTGAG |
| **ABCA8 forward** | GGGGTTTGCTCTATGCTGGT |
| **ABCA8 reverse** | CAGTGAGGAGGAACACGACC |
| **ABCA9 forward** | ATGCAGAGACCCATGGATGAAC |
| **ABCA9 reverse** | ATGTTTGCTGACCCACGCTC |
| **ABCG1 forward** | GGTCGCTCCATCATTTGCAC |
| **ABCG1 reverse** | TGTGGTAGGTTGGGCAGTTC |
| **TAP2 forward** | TCAAGGGGCTGACGTTTACC |
| **TAP2 reverse** | AGAACCGGAGAACAGCACAG |
| **BSCL2 forward** | ATGGTCAACGACCCTCCAGTA |
| **BSCL2 reverse** | GCTGACTGTCGGCATATAGGAA |

| **siRNAs** | **Target sequences** |
| --- | --- |
| **si-h-FOXS1** | CACTCAACGAGTGCTTTGT |
| **si-h-ABCA1** | CCAGCCAGCTTTGTCGTAT |
| **si-h-ABCG1** | ATCGTACTCGGGATTTTCT |
| **si-h-BSCL2** | CTACTCTGACTACTCGAGTAG |

**Table S3. Comparison of echocardiographic, hemodynamic and metabolic parameters in Apoe^-/-^ mice and Apoe^-/-^Foxs1^-/-^ mice fed with HFD.**

| Parameters | Apoe^-/-^ | Apoe^-/-^Foxs1^-/-^ | *P* value |
| --- | --- | --- | --- |
|  | (n = 10) | (n = 10) |  |
| BW before, g | 21.83 ± 1.64 | 20.65 ± 1.48 | 0.139 |
| BW after, g | 41.35 ± 2.41 | 40.58 ± 2.38 | 0.7127 |
| Heart rate, bpm | 518.6 ± 21.6 | 527.3 ± 30.2 | 0.3586 |
| EF, % | 51.67 ± 8.71 | 50.47 ± 7.14 | 0.741 |
| LVEDd, mm | 3.18 ± 0.21 | 3.23 ± 0.27 | 0.3754 |
| FS, % | 45.39 ± 2.42 | 46.87 ± 2.69 | 0.1238 |
| MAP, mmHg | 81.64 ± 9.61 | 83.72 ± 8.86 | 0.8029 |
| Glucose, mmol/L | 9.75 ± 1.89 | 10.18 ± 2.08 | 0.6891 |
| TC, mmol/L | 28.85 ± 3.47 | 29.64 ± 4.21 | 0.8570 |
| LDL, mmol/L | 27.12 ± 2.87 | 26.75 ± 3.43 | 0.9802 |
| TG, mmol/L | 2.09 ± 0.54 | 2.14 ± 0.62 | 0.5246 |
| HDL, mmol/L | 1.16±0.24 | 1.24±0.32 | 0.6381 |

Abbreviations: Apoe^-/-^, apolipoprotein E–deficient. BW, body weight. EF, ejection fraction. FS, functional shortening. LVEDd, left ventricular end-diastolic diameter. MAP, mean arterial blood pressure. TC, triglyceride. LDL, low density lipoprotein. TG, total cholesterol. Values are mean ± standard deviation (SD) or %. Statistical differences were determined using two-tailed unpaired Student’s t-test.

**Table S4. Comparison of echocardiographic, hemodynamic and metabolic parameters in Apoe^-/-^ mice and Apoe^-/-^+rmBscl2 mice fed with HFD.**

| Parameters | Apoe^-/-^ | Apoe^-/-^+rmBscl2 | *P* value |
| --- | --- | --- | --- |
|  | (n = 10) | (n = 10) |  |
| BW before, g | 20.43 ± 1.28 | 20.86 ± 1.67 | 0.344 |
| BW after, g | 41.85 ± 2.36 | 40.97 ± 2.71 | 0.4504 |
| Heart rate, bpm | 522.6 ± 28.4 | 530.8 ± 29.7 | 0.8923 |
| EF, % | 52.21 ± 8.00 | 50.12 ± 9.74 | 0.606 |
| LVEDd, mm | 3.13 ± 0.18 | 3.21 ± 0.25 | 0.2074 |
| FS, % | 46.31 ± 2.52 | 47.31 ± 2.16 | 0.1123 |
| MAP, mmHg | 82.39 ± 9.27 | 81.79 ± 10.14 | 0.6785 |
| Glucose, mmol/L | 10.21 ± 2.09 | 10.72 ± 2.67 | 0.1755 |
| TC, mmol/L | 27.18 ± 2.94 | 29.37 ± 3.18 | 0.3931 |
| LDL, mmol/L | 26.32 ± 3.64 | 26.96 ± 3.39 | 0.3010 |
| TG, mmol/L | 2.01 ± 0.48 | 2.18 ± 0.71 | 0.5931 |
| HDL, mmol/L | 1.08±0.27 | 1.19±0.33 | 0.2520 |

Abbreviations: Apoe^-/-^, apolipoprotein E–deficient. BW, body weight. EF, ejection fraction. FS, functional shortening. LVEDd, left ventricular end-diastolic diameter. MAP, mean arterial blood pressure. TC, triglyceride. LDL, low density lipoprotein. TG, total cholesterol. Values are mean ± standard deviation (SD) or %. Statistical differences were determined using two-tailed unpaired Student’s t-test.

**Excel File S1**. Analyzed results of the RNA sequencing data.
