# Supplementary material for: Oxidized LDL-induced FOXS1 mediates cholesterol transport dysfunction and inflammasome activation to drive aortic valve calcification
Source: Cardiovasc Res. 2025 Sep 24;121(12):1941–55. doi: 10.1093/cvr/cvaf159 (PMC12551391; doi:10.1093/cvr/cvaf159)

Unedited gel for each representative cropped gel within the manuscript

Figure 1D

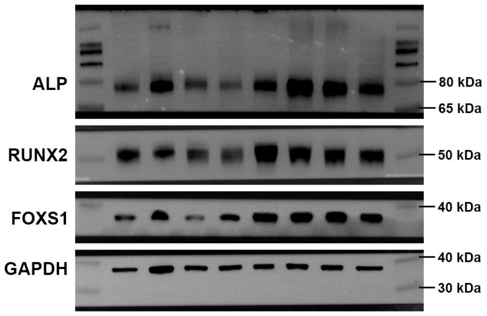

Figure 1I

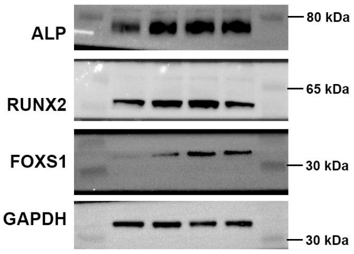

Figure 1J

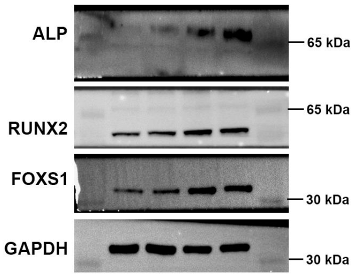

Figure 2A

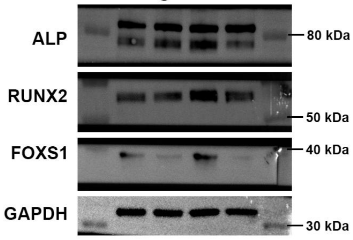

Figure 2C

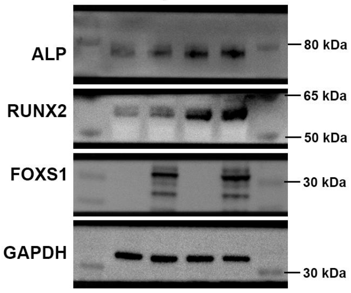

Figure 4H

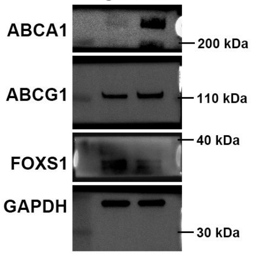

Figure 4I

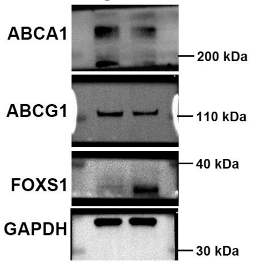

Figure 4J

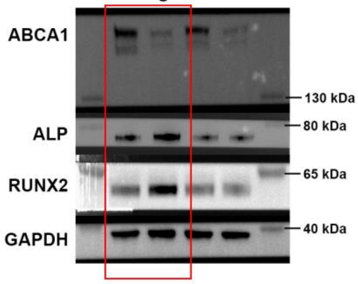

Figure 4K

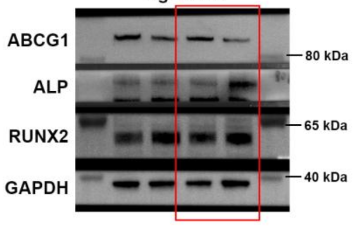

Figure 5D

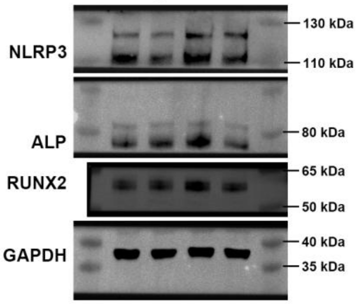

Figure 5C

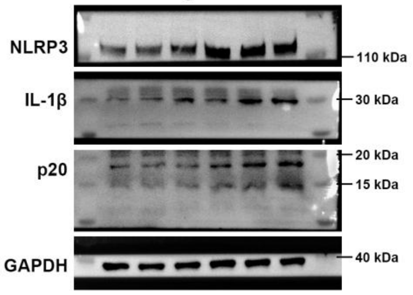

Figure 7G

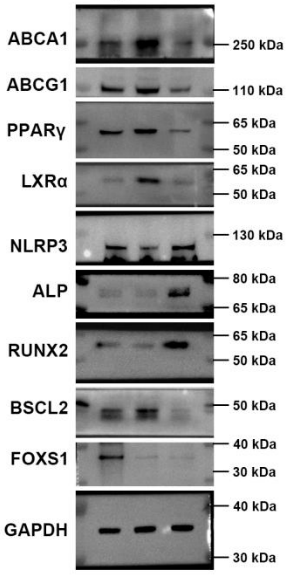

Figure 6A

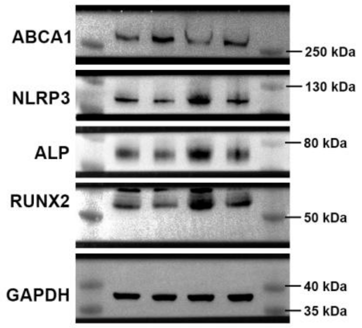

Figure S4

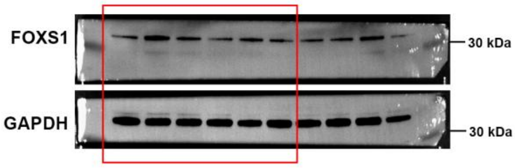

Figure S5

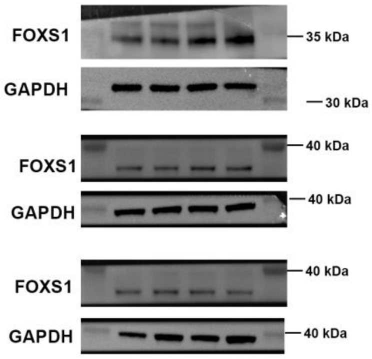

Figure S6

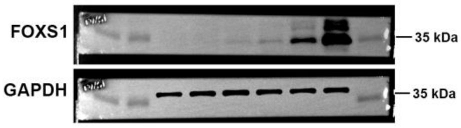

Figure S12

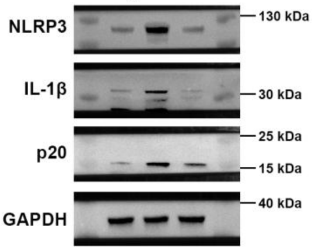

Figure S13

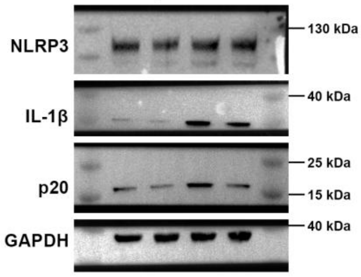

Figure S14

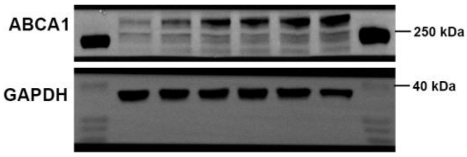

Supplement: cvaf159_Supplementary_Data [file cvaf159_supplementary_data.zip › Unedited gel.pdf]
